# Catechol Siderophores from a Mangrove-Derived Bacteria *Serratia marcescens* F2-2 and Their Cytotoxic Activity

**DOI:** 10.3390/md23060241

**Published:** 2025-05-30

**Authors:** Gang Zhang, Xunming Wang, Xingwang Zhang, Lin Ye, Longyang Ke, Shimin Fan, Xuan Hong, Guoqiang Li, Bingye Yang, Lianzhong Luo

**Affiliations:** 1 Engineering Research Center of Marine Biopharmaceutical Resource of Fujian Province University, Xiamen Medical College, Xiamen 361023, China; zg@xmmc.edu.cn (G.Z.); w729057918@outlook.com (X.W.); yelin311728@gmail.com (L.Y.); long38763@163.com (L.K.); fan5himin@163.com (S.F.); 2 Xiamen Key Laboratory of Marine Medicinal Natural Product Resources, Xiamen Medical College, Xiamen 361023, China; hx@xmmc.edu.cn; 3 State Key Laboratory of Microbial Technology, Shandong University, Qingdao 266237, China; zhangxingwang@sdu.edu.cn; 4 Key Laboratory of Marine Drugs, Ministry of Education, School of Medicine and Pharmacy, Ocean University of China, Qingdao 266003, China; liguoqiang@ouc.edu.cn

**Keywords:** *S. marcescens*, serratiochelins, cytotoxicity, apoptosis

## Abstract

*Serratia marcescens* is a common Gram-negative and facultative anaerobic bacillus that produces serratiochelins with several bioactivities. In this study, four catechol siderphores (**1**–**4**), including two new ones named serratiochelins E (**1**) and F (**2**), were obtained from the fermentation of a mangrove-derived bacterium, *S*. *marcescens* F2-2. The structures were elucidated with various spectroscopic methods such as NMR and HR-ESI-MS. Absolute and geometric configurations of the new compounds were established by employing quantum NMR calculations in conjunction with DP4+ probability analysis, ECD calculations, and the advanced Marfey’s method. The bioactivity test showed that serratiochelin B (**3**) displayed weak but selective cytotoxicity against HepG2 cancer cells with an IC_50_ of 50.6 μmol/L and could trigger apoptosis through both Bcl-2/Bax/caspase-3 and Fas/FasL/caspase-8 signaling pathways. These findings deepen the understanding of siderophores of *S. marcescens* and provide a lead for research on anti-liver cancer drugs.

## 1. Introduction

Serratiochelins are a class of catechol-type siderophores produced by the genus *Serratia*, particularly *Serratia marcescens* [1,2]. Functionally, siderophores like serratiochelins could form high-affinity Fe^3+^ complexes to scavenge iron, enter the bacterial periplasm via TonB-ExbBD-dependent transporters after being recognized by outer membrane receptors, and are crucial for bacterial growth under iron-limited conditions [3]. For example, in *S. marcescens*, mutants lacking the serratiochelin gene cluster show significantly reduced iron-chelating ability and decreased growth under iron limitation, and were outcompeted by wild-type strains in a bacteremia mouse model, highlighting their role in bacterial pathogenesis [4].

As typical catecholate-type iron chelators, serratiochelins have a backbone of two 2,3-dihydroxybenzoyl groups: a threonyl group which sometimes reacts with the 2,3-dihydroxybenzoyl to form a methyl oxazolin-4-carbonyl group and a 1,3-propanediamine group, which forms structures that facilitate bacterial iron acquisition in iron-deficient environments with high affinity. Up to now, a few serratiochelin analogs (serratiochelins A–D) have been reported in the literature [1,5]. Interestingly, these serratiochelins have exhibited some biological activities. For instance, serratiochelins A, B, and D exhibited xenosiderophoric activity by promoting the growth of *Staphylococcus aureus* [1], *Acinetobacter baumannii* [1,6], or *Mycobacterium tuberculosis* [1,6] in iron-limited media and thus have the potential to be developed into sideromycins conjugated to other antibiotics using a Trojan horse strategy [7]. Additionally, serratiochelin A displayed an inhibitory effect against *S. aureus* [8] and demonstrated cytotoxic properties on human monocytic leukemia cells (THP-1) [6], human embryonic kidney cells (HEK-293) [6], human lung fibroblast cells (MRC5) [8], and human melanoma cells (A2058) [8]. Serratiochelin A also showed potent DPPH radical-scavenging activity [9]. These findings suggest serratiochelins could serve as lead compounds for drug research.

**Figure 1:**
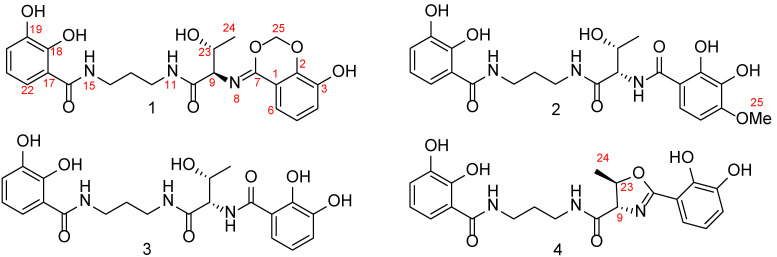
Structures of serratiochelins E (**1**), F (**2**), B (**3**), and A (**4**) from *S. marcescens* F2-2.

Lung and liver cancers are among the top causes of cancer-related deaths [10,11]. Liver cancer, for instance, presents significant challenges in drug development, including its complex mechanisms, the risk of drug-induced liver toxicity, and the difficulty in achieving specific targeting. Recently, natural products have garnered attention for their ability to selectively regulate enzyme activities involved in carcinogenesis [12]. This selective regulation can influence early hepatocarcinogenesis and inhibit liver cancer formation, making natural products important for related drug research [13]. During our exploration of active natural products from marine organisms, a bacterial strain (*S. marcescens* F2-2) isolated from the mangrove mud of Zhangjiangkou Mangrove National Nature Reserve of China attracted our attention due to its characteristic production of serratiochelins, as detected by LC-MS analysis. Consequently, we investigated the fermentation products of F2-2 and successfully obtained four serratiochelins (**1**–**4,** Figure 1) and interestingly, there were two new ones (serratiochelins E and F, **1**–**2**). Herein, we reported the fermentation, extraction, separation, structural identification, and cytotoxic activity evaluation of **1**–**4**. The preliminary mechanism of serratiochelin B (**3**) inducing the apoptosis of HepG2 cells was also experimentally evaluated. The results confirmed that **3** could serve as a lead for liver cancer drug research.

## 2. Results and Discussion

### 2.1. Structural Elucidation of Serratiochelins E and F

The EtOAc extract of the rice fermentation of *S. marcescens* F2-2 was subjected to extensive column chromatography over octadecylsilane (ODS) and Sephadex LH-20. The subfractions were purified through the semi-preparative HPLC, which yielded compounds **1**–**4**. By comparing the NMR and MS data with references, two previously reported compounds were identified as serratiochelin B (**3**) [5] and serratiochelin A (**4**) [2], respectively.

**Figure 2:**
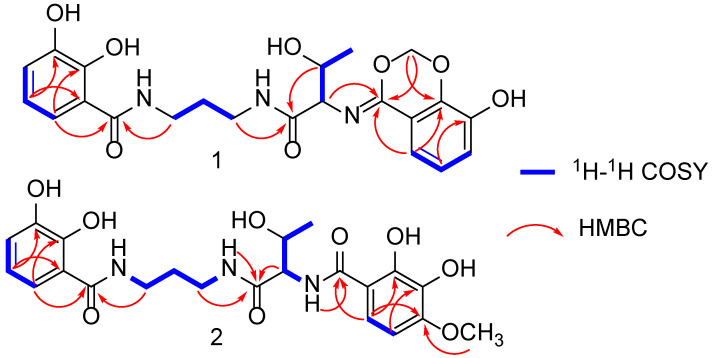
The key COSY (bold) and HMBC (arrow) correlations of compounds **1** and **2**.

The HR-ESI-MS spectrum of compound **1** exhibited a [M-H]^−^ ion peak at *m*/*z* 458.1573 (calculated for 458.1569), indicative of a molecular formula C_22_H_25_N_3_O_8_ with 12 degrees of unsaturation. The ^13^C NMR spectrum revealed 12 aromatic carbon signals among *δ*_C_ 116.7 and 150.3 (Table 1), pointing to two aromatic rings. A propane 1,3-diamine moiety was identified via the ^1^H-^1^H COSY spectrum, which showed a CH_2_-CH_2_-CH_2_ fragment (Figure 2 and Figure S5). Specifically, correlations of *δ*_H_ 1.84 with *δ*_C_ 30.2, *δ*_H_ 3.31 with *δ*_C_ 37.7, and*δ*_H_ 3.44 with *δ*_C_ 37.8 in the HMQC spectrum (Figure S6) further supported this. Additionally, a CH_3_–CH–CH spin-coupled system detected in the ^1^H-^1^H COSY spectrum (Figure S5), along with corresponding carbon signals at *δ*_C_ 20.3, 67.4, and 62.2, confirmed the presence of a threonyl fragment in compound **1**. In the HMBC spectrum (Figure 2 and Figure S7), correlations of *δ*_H_ 7.22 and 3.31 with *δ*_C_ 171.7, and *δ*_H_ 3.44 and 5.08 with another carbonyl carbon at *δ*_C_ 171.7, established that the propane1,3-diamine moiety is connected to a 2,3-dihydroxybenzoyl group and a threonine residue via two secondary amide bonds. Furthermore, a sp^3^-hybridized methylene signal (*δ*_H_ 5.64, 5.55) in the HMBC spectrum correlated with *δ*_C_ 148.2 and 165.3 (Figure 2). Also, the α-H in the threonine residue (*δ*_H_ 5.08) and H-6 in the benzene ring (*δ*_H_ 7.38) correlated with *δ*_C_ 165.3 (Figure 2). These data collectively suggested the presence of a six-membered 1,3-dioxane ring in **1**, as depicted in Figure 1.

**Figure 3:**
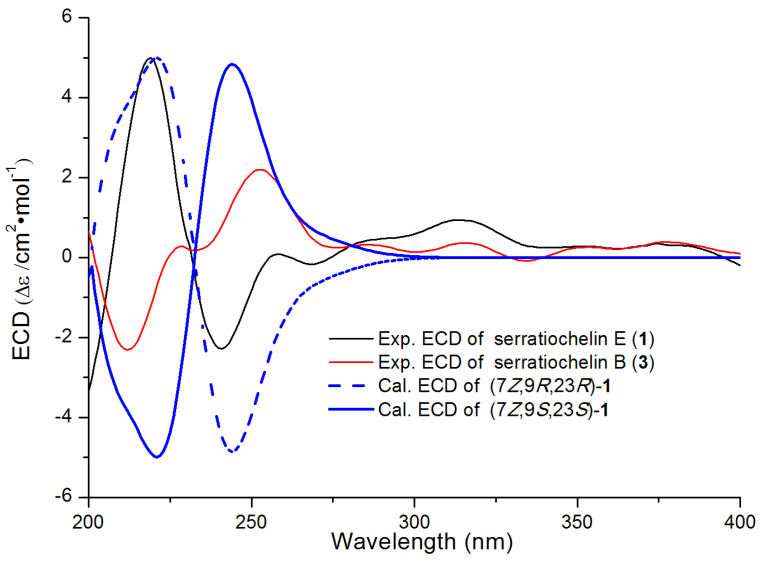
Experimental ECD spectra of **1**, **3** and calculated ECD spectra of (7*Z*,9*R*,23*R*)-**1** and (7*Z*,9*S*,23*S*)-**1.**

The absolute configuration of threonine residue and geometric configuration of the ∆^7(8)^ double bond were determined by NMR calculations combined with regression analysis, DP4+ analysis, and ECD calculations. The NMR data calculations [14] were performed for the four isomers, namely (7*Z*,9S^*^,23R^*^)-**1** (**1a**), (7*E*,9S^*^,23R^*^)-**1** (**1b**), (7*E*,9R^*^,23R^*^)-**1** (**1c**), and (7*Z*,9R^*^,23R^*^)-**1** (**1d**) (Figure S8). The regression analysis indicated that for both the ^13^C NMR and ^1^H NMR, the correlation coefficient of (7*Z*,9R^*^,23R^*^)-**1** (**1d**) was higher than other isomers, leading to the conclusion that the calculated ^13^C NMR and ^1^H NMR data of **1d** were closest to the experimental data of **1.** Also, the isomer (7*Z*,9R^*^,23R^*^)-**1** (**1d**) exhibited a DP4+ probability of 99.89% (Figure S9). Based on the above results, it can be inferred that the ∆^7(8)^’s geometric configuration was *Z*-form and the relative configuration of the threonine residue was 9R^*^,23R^*^. Subsequently, the ECD spectrum calculation of (7*Z*,9*R*,23*R*)-**1** (**1c**) was carried out at the B3LYP/6-311G (d, p) level [15]. It was found that the calculated ECD spectrum of (7*Z*,9*R*,23*R*)-**1** was aligned with that of the experimental spectrum (Figure 3). Thus, the absolute configuration of the threonine moiety was determined to be 9*R*,23*R*. Based on these findings, the structure of compound **1** was elucidated as 2,3-dihydroxy-*N*-(3-((2*R*,3*R*)-3-hydroxy-2-(((*Z*)-8-hydroxy-4*H*-benzo[*d*][1,3]dioxin-4-ylidene)amino)butanamido)propyl)benzamide and named serratiochelin E.

The HR-ESI-MS spectrum of compound **2** (serratiochelin F) exhibited a peak at *m*/*z* 476.1677 [M-H]⁻ (calculated for 476.1674), indicative of a molecular formula of C_22_H_27_N_3_O_9_ with 11 degrees of unsaturation. A comparison of the NMR data of **2** with those of co-isolate **3** [5] revealed that these two compounds are structurally similar. The ¹H-¹H COSY cross-peaks between H-5 and H-6 (Figure 2), along with HMBC correlations from H-6 to C-10 and from H_3_-25 to C-4, established that **2** has a methoxyl group at position C-4, as depicted in Figure 1. The absolute configuration of the threonine residue was determined using the advanced Marfey’s method [16]. Amino acid standards of L-threonine, D-threonine and L-*allo*-threonine, and the hydrolysate of **2,** were derivatized with L-N^α^-(5-fluoro-2,4-dinitrophenyl)-L-leucinamide (L-FDLA). The comparison of the retention time of FDLA derivatives from the hydrolysate of **2** with those of the amino acid standards via UPLC-MS confirmed the L configuration for the Thr residue (Figure S16). Consequently, the structure of **2** was identified as 2 N-((2*S*,3*R*)-1-((3-(2,3-dihydroxybenzamido)propyl)amino)-3-hydroxy-1-oxobutan-2-yl)-2,3-dihydroxy-4-methoxybenzamide.

**Scheme 1:**
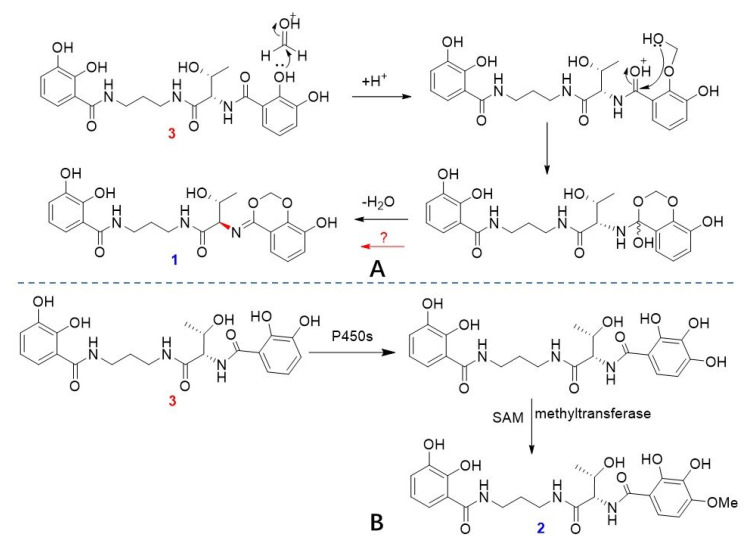
Relatively reasonable biosynthesis pathways for **1** (**A**) and **2** (**B**).

The relatively reasonable biosynthesis for serratiochelins E (**1**) and F (**2**) is proposed herein. With serratiochelin B (**3**) as a precursor, an electrophilic addition reaction with formaldehyde followed by dehydration led to the formation of **1** (Scheme 1A) [17]. Additionally, under the promotion of cytochrome P450s [18], **3** underwent a hydroxylation at the C-4 position. Subsequently, a methylation reaction was catalyzed by SAM-dependent methyltransferases (MTs) [19], which yielded **2** (Scheme 1B). However, the configuration reversal at C-9 requires further investigation.

### 2.2. Cytotoxic Effects of Compounds **1**–**4**

#### 2.2.1. Inhibition and Selectivity of Tumor Cell Proliferation by **3**

All isolates (**1**–**4**) were tested for antiproliferative activities against A549 lung and HepG2 liver cancer cells using the CCK-8 assay [20]. However, the results showed that only serratiochelin B (**3**) displayed weak but selective inhibitory activity against HepG2 cell lines, and it was speculated that HepG2 showed a certain sensitivity to the N-(2,3-dihydroxybenzoyl)threoninamidyl moiety presented in the serratiochelins. The IC50 values of the positive control (doxorubicin) were 2.35 μmol/L for HepG2 cells and 1.12 μmol/L for A549 cells, respectively. It was speculated that HepG2 showed a certain sensitivity to the N-(2,3-dihydroxybenzoyl)threoninamidyl moiety presented in the serratiochelins.

**Figure 4:**
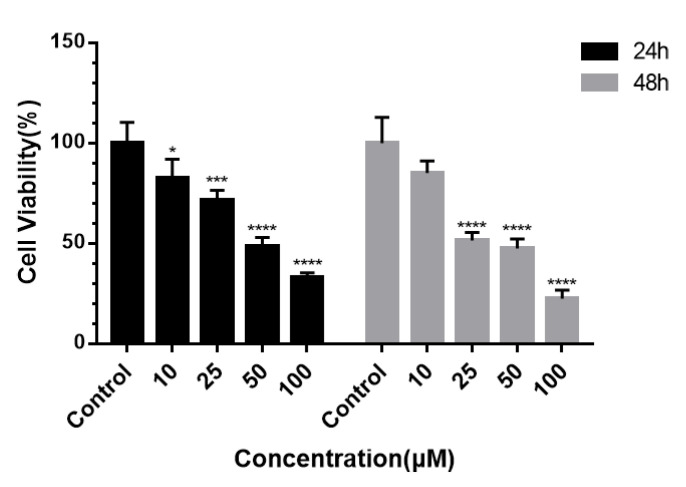
Inhibitory effects of **3** on the proliferation of HepG2 tumor cells compared with the control group, * *p* < 0.05, *** *p* < 0.0002, **** *p* < 0.0001.

To further investigate the inhibitory effects of **3** on the proliferation of HepG2 cells, HepG2 cells were treated with various concentrations of **3** (0, 10, 25, 50 and 100 μmol/L) for 24 and 48 h, followed by assessment of cell viability. The results, as shown in Figure 4, demonstrated that **3** exerted a concentration-dependent inhibitory effect on HepG2 cells within the concentration assessed, with a more pronounced effect was observed at 48 h. At 24h, the IC_50_ value was 50.6 μmol/L. The IC_50_ values of the positive control (doxorubicin) were 2.35 μmol/L for HepG2 cells and 3.12 μmol/L for A549 cells, respectively.

HepG2 cells were treated with 50 μmol/L of **3** for 24 h and then stained with acridine orange (AO) and propidium iodide (PI) to assess the ratio of live to dead cells and to observe whether the apoptosis occurred [21], thereby verifying the antiproliferative activity of **3** against HepG2 tumor cells. As shown in Figure S18, under fluorescence microscopy with an excitation wavelength of 488 nm, the proportion of HepG2 cells stained brightly green by AO decreased after treatment with **3**. Moreover, chromatin condensation, a hallmark of apoptosis [22], was observed. These results indicated that **3** significantly inhibits the proliferation of HepG2 cells at the tested concentration and that apoptosis was involved in this process.

#### 2.2.2. Modulation of Apoptosis-Related Protein Expression by **3**

**Figure 5:**
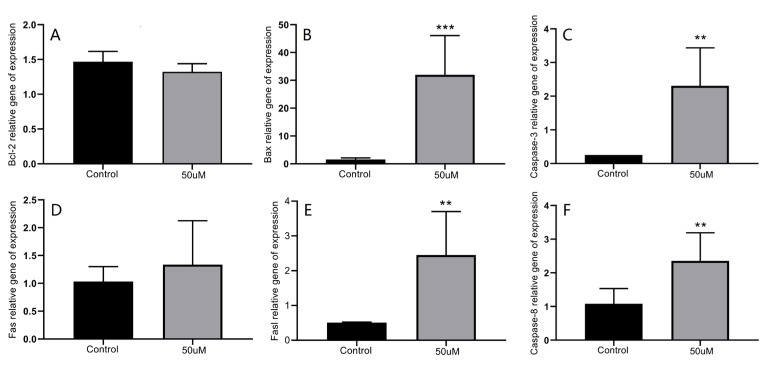
Serratiochelin B regulated the expression of apoptosis-related proteins in HepG2 cells at the dose of 50 μmol/L. (**A**–**F**) Quantitative analysis of the relative RT-qPCR expression levels of Bcl-2, Bax, caspase-3, Fas, FasL, and caspase-8, respectively. All values are expressed as mean±SD, ** *p* < 0.005, *** *p* < 0.0002 versus the control group.

The mitochondrial apoptosis pathway is a crucial signaling cascade in apoptosis, which is primarily regulated by the Bcl-2 family of proteins [23] and caspase-3, a key downstream effector protease, is pivotal in this pathway [24]. Bcl-2, an antiapoptotic factor, inhibits apoptosis by binding to proapoptotic factors or preventing the release of apoptotic proteins from mitochondria to the cytoplasm. Conversely, Bax, a proapoptotic factor, promotes apoptosis. The balance between Bcl-2 and Bax, reflected by their ratio, is a critical indicator of whether apoptosis has been initiated [25]. In the present study, the expression level of Bcl-2 in HepG2 cells remained unchanged when treated with 50 μmol/L of **3** for 24 h, while Bax expression significantly increased (*p* < 0.0002). Additionally, the expression level of caspase-3 also rose significantly (*p* < 0.005) (Figure 5, A-C). These findings suggested that **3** can upregulate the Bax/Bcl-2 ratio and increase the expression of activated caspase-3, thereby inducing apoptosis in HepG2 cells via the mitochondrial pathway.

The Fas/FasL death receptor-mediated pathway is another significant apoptotic pathway [26]. It is initiated by the binding of FasL to the cell surface receptor Fas. The activated Fas receptor, which contains a death domain (DD) in its cytoplasmic segment, specifically interacts with the Fas-associated death domain protein (FADD) to form a death-inducing signaling complex (DISC) [27]. This complex subsequently activates caspase-8, leading to apoptosis [28]. In our experiments, expression levels of FasL and caspase-8 were significantly elevated (*p* < 0.005), while the Fas expression remained unchanged (Figure 5D–F). These results indicated that **3** likely induced apoptosis in HepG2 cells through the Fas/FasL death receptor pathway.

## 3. Materials and Methods

### 3.1. Bacterial Materials

The bacterial strain *S. marcescens* F2-2, was sourced from the mangrove mud of Zhangjiangkou Mangrove National Nature Reserve of China and was identified by the 16S rRNA gene sequence analysis. The gene sequence, colony morphology, and phylogenetic tree were listed in Figure S1. The voucher strain was preserved at Guangdong Microbial Culture Collection Center and the preservation number is GDMCC No.65860.

### 3.2. General Experimental Procedures

The physical data of **1**–**4** were obtained using the following instruments: a UV-2600 spectrometer (Shimadzu, Kyoto, Japan) for the UV spectra; a Bruker TENSOR 27 FTIR Spectrometer (Bruker, Mannheim, Germany) for the IR spectra; a Chirascan circular dichroism spectrometer (Applied Photophysics, Leatherhead, UK) for specific rotations; an Avance-NEO 500 MHz NMR Spectrometer (Bruker, Billerica, MA, USA) for the NMR data (^1^H-NMR: 500 MHz, ^13^C-NMR: 125 MHz) using solvent residual peaks (CD_3_OD: 3.31/49.0 and DMSO-*d*_6_: 2.50/39.5 ppm) as internal standards; a LC-MS 8050 of triple-quadrupole liquid chromatography–mass spectrometry system (Shimadzu, Kyoto, Japan) system for the detection of the FDLA derivatives of threonine isomers; and a qQ Exactive hybrid quadrupole–orbitrap mass spectrometer (Thermo Fisher Scientific Inc, Waltham, MA, USA) for the HR-ESI-MS data. An Agilent 1260 HPLC (Agilent Technologies, Santa Clara, CA, USA) equipped with a C_18_ column (5 µm, 10 × 250 mm, Reprosil Gold 120, Ammerbuch, Germany) was used for the HPLC separation. For the microscopic analysis, a confocal laser scanning microscope (Leica, Mannheim, Germany) was employed. Quantitative PCR was performed using the 7500 Fast system (Applied Biosystems, Foster City, CA, USA). The nucleic acid concentration was measured with a Q5000 nanodrop spectrophotometer (Quawell Technology, San Jose, CA, USA). The human lung adenocarcinoma epithelial cells (A549) and human liver carcinoma cells (HepG2) used in the experiment were purchased from Pricella Biotechnology Co., Ltd., Wuhan, China. Cell cultures were maintained in a CO_2_ incubator from Thermo Fisher Scientific, Waltham, MA, USA. The reagents included a CCK-8 assay kit from Beijing Lanjieke Technology, a cDNA synthesis kit from TaKaRa Bio, SYBR Green Master Mix from Yeasen Biotechnology, and an Annexin-V-FLUOS staining kit from Roche, Shanghai, China. AO/PI double staining was performed using a kit from Bestbio Biotechnology, Shanghai, China, and RNA extraction was carried out with TRIzol reagent from Thermo Fisher Scientific, Waltham, MA, USA. All other reagents used were of analytical grade.

### 3.3. Fermentation, Extraction, and Purification

The activated F2-2 strain was taken from a sabouraud dextrose agar (SDA) plate and inoculated into 100 mL of SDA liquid medium. The culture was then incubated in a constant-temperature shaking incubator at 28 °C and 150 rpm for 3 days. Subsequently, 1000 mL Erlenmeyer flasks were used as fermentation vessels in which the rice solid mediums were prepared (180 g of rice with 240 mL of distilled water) and sterilized at 121 °C for 20 min, followed by cooling. Under aseptic conditions, 10 mL of F2-2 seed liquid was transferred to the fermentation flask, and the static fermentation was carried out at 25 °C for 1 month. After fermentation, the F2-2 rice culture was crushed, and then soaked in six times the volume of ethyl acetate. Ultrasonic extraction was performed for 30 min, followed by filtration to obtain a deep red filtrate. The residue was subjected to another round of extraction with six times the volume of ethyl acetate using the same method. The combined extracts from both extractions were concentrated using a rotary evaporator at 40 °C under reduced pressure to obtain a deep red paste.

The sample was subjected to a ODS (120A, 50 μm, YMC, Kyoto, Japan) column and eluted with 10%, 30%, 50%, 70%, 90% and 100% methanol in water, respectively. The elution solvent was evaporated under reduced pressure at 40 °C using a rotary evaporator, yielding corresponding elution fractions F1-F6. The HPLC analysis revealed that the components eluted with 30% methanol (F2) were similar to those eluted with 50% methanol (F3). Hence, these fractions were combined and subjected to a Sephadex LH-20 gel (GE Healthcare Bio-Sciences, Uppsala, Sweden) column chromatography using pure methanol as the eluent and six subfractions, and six subfractions of F2.1–F2.6 were obtained. The F2.4 fraction was subjected to the semi-HPLC preparative separation and the separation condition was as follows: isocratic elution with methanol–water (55% methanol containing 0.1% formic acid), a flow rate of 1.5 mL/min, injection volume of 50 μL, and a detection wavelength of 210 nm. Then, compounds **3** (19.9 mg) and **2** (4.2 mg) were obtained. For the F2.5 fraction, the sample was also subjected to semi-HPLC equipment and the separation condition was the same as those of F2.4. Consequently, **4** (8.6 mg) and **1** (5.1 mg) were yielded. The purities of serratiochelins A (**4**), B (**3**), and F (**2**) were 87.6%, 98.5%, and 94.7%, respectively. Although serratiochelin E (**1**) was prepared as a single peak, the HPLC analysis showed two peaks, with the main peak (serratiochelin E) having a purity of 60.0%.

Serratiochelin E (**1**): white powder; ${\left[\alpha \right]}_{\;D}^{20}$ +66.3 (c 0.10, CH_3_OH); UV (CH_3_OH) *λ*_max_ (DAD) 252, 318 nm; ECD (MeOH) *λ*_max_ (Δε) 218 (+1.27), 242 (−0.70); IR (film) *ν*_max_ 3268, 3243, 2976, 2875, 1652, 1591, 1257, 1058, 751 cm^−1^ HR-ESI-MS at *m*/*z* 458.1573 [M-H]^−^ (calculated for C_22_H_24_N_3_O_8_^−^, 458.1569); ^1^H NMR (CD_3_OD, 500 MHz) and ^13^C NMR (CD_3_OD, 125 MHz), see Table 1.

Serratiochelin F (**2**): white powder; ${\left[\alpha \right]}_{\;D}^{20}$ −4.2 (c 0.10, CH_3_OH); UV (CH_3_OH) *λ*_max_ (DAD) 253, 317 nm; ECD (MeOH) *λ*_max_ (Δε) 210 (−1.04), 252 (+0.42); IR (film) *ν*_max_ 3351, 3322, 2924, 2854, 1636, 1598, 1258, 1056 cm^−1^; HR-ESI-MS at *m*/*z* 476.1677 [M-H]^−^ (calculated for C_22_H_26_N_3_O_9_^−^, 476.1674); ^1^H NMR (DMSO-*d*_6_, 500 MHz) and ^13^C NMR (DMSO-*d*_6_, 125 MHz), see Table 1.

### 3.4. Determination of the Absolute Configuration of Threonine Residue [29]

Firstly, 0.05 mg of **2** was dissolved in 0.5 mL of 6 N HCl, respectively, and heated at 115 °C for 2 h, and then cooled immediately in an ice bath for 5 min, and the mixture was evaporated to dryness under a stream of nitrogen. After that, 0.1mg L-FDLA (Nalpha-(5-Fluoro-2,4-dinitrophenyl)-L-leucinamide, Tokyo Chemical Industry, Tokyo, Japan), 100 µL of acetonitrile, and 100 µL of 1% sodium carbonate solution were added to the hydrolysate, and the reaction was allowed to proceed at 45 °C for 1 h. After the reaction, 10 µL of 1 M HCl was added to terminate the reaction. FDLA derivatives of L-threonine (Tokyo Chemical Industry, Tokyo, Japan), L-*allo*-threonine (Tokyo Chemical Industry, Tokyo, Japan), and D-threonine (Angon Biotech, Shanghai, China) standards were prepared via the same procedure except for the hydrolysis procedure.

Next, 1 µL of the reaction mixture was analyzed by the LC-MS system and the analysis conditions were as follows: gradient elution of 30% to 100% acetonitrile in water with 0.1% formic acid for 10 min and a flow rate of 0.25 mL/min; the SIM model was used to detect molecular weights of FDLA–threonine derivatives (414 [M+H]^+^) in the positive-ion condition. The absolute configuration of threonine was determined by comparing the retention time with those of L-threonine, L-*allo*-threonine, and D-threonine standards.

### 3.5. NMR and ECD Calculations

The conformational analyses for compound **1** were performed via Yinfo Cloud Platform using a systematic algorithm by Confab [30] with an MMFF94 force field with an RMSD threshold of 0.5 Å and an energy window of 7 kcal/mol. The theoretical calculations were carried out using Gaussian 09. The conformers of **1** were optimized at B3LYP/6-31G(d) in gas phase. ECD calculations were conducted at B3LYP/6-311G(d,p) level in methanol with IEFPCM model using time-dependent density functional theory (TD-DFT). NMR calculations were carried out following the protocol adapted from Michael et al. [31] using the Gauge-Including Atomic Orbitals (GIAOs) method at mPW1PW91/6-311+G(2d,p) level in MeOH simulated by the IEFPCM model. NMR chemical shift values were averaged according to Boltzmann distribution and fitted to the experimental values by linear regression. To confirm the conclusions of NMR calculations, DP4+ analysis was also performed [14].

### 3.6. Toxicity Detection

Cytotoxic effects of **1**–**4** on A549 and HepG2 tumor cell lines were performed using tetrazolium-8-[2-(2-methoxy-4-nitrophenyl)-3-(4-nitrophenyl)-5-(2,4-disulfophenyl)-2H-tetrazolium] monosodium salt (CCK-8) assay [20]. The basal culture medium for both HepG2 and A549 cells was the same. It consisted of high-glucose Dulbecco’s modified Eagle’s medium (DMEM, Pricella Biotechnology Co., Ltd. Wuhan, China) and was supplemented with 10% fetal bovine serum (FBS) and 1% penicillin–streptomycin. Tumor cells in logarithmic growth phase were seeded into 96-well plates at a density of 5 × 10^5^ cells/mL, with 200 μL per well and 3 replicates per group. Edge wells were filled with 100 μL of 1 × PBS or 0.9% NaCl solution. After incubation at 37 °C with 5% CO_2_ for 4–8 h to allow cell adhesion, the supernatant was removed. Blank wells received 100 μL of basic culture medium, while experimental wells were treated with serratiochelin solutions at a concentration of 100 μmol/L. The plates were then incubated for 24 h and 48 h. After incubation, the supernatant was removed, and 100 μL of 10% CCK-8 solution was added to each well, followed by incubation for 30 min. Absorbance was measured at 450 nm using a microplate reader to assess the cell viability. If cell viability was less than 50%, additional experiments with concentrations of 10, 25, 50 and 100 μmol/L were performed to determine the IC_50_ of the tested compound. Doxorubicin (Sigma-Aldrich) was used as the positive control.

### 3.7. Detection of Apoptosis Using AO/PI Double Staining

The apoptotic effects of **3** on HepG2 cells were tested using the AO/PI double staining method [32]. HepG2 cells were seeded into a 96-well plate following the method described in Section 3.6 for the CCK-8 assay. Cells were first collected from a 96-well plate after 24 h of treatment. The medium with cells was transferred to centrifuge tubes, centrifuged at 1500 rpm for 3 min, and the supernatant was discarded to collect floating cells. The adherent cells in the wells were washed twice with 1×PBS, ensuring that no cell loss occurred. Next, the AO/PI stain solution was added to the wells, mixed with the collected floating cells, and incubated at 37 °C for 15 min. After incubation, the staining solution was removed, and the cells were washed twice with pre-warmed basic culture medium. The stained cells were then observed under a fluorescence microscope with 488 nm excitation light, and images were captured. For result analysis, normal cells exhibited uniform green nuclei, apoptotic cells showed condensed chromatin and fragmented nuclei with dense green fluorescence, and necrotic cells appeared as strong red fluorescence.

### 3.8. RT-qPCR Detection of the Effect of **3** on the Gene Expression of Apoptosis-Related Factors and Signaling Pathway Proteins

To investigate the effects of **3** on apoptosis-related factors and signaling pathway proteins in HepG2 cells, RT-qPCR analysis was employed [33]. HepG2 cells were cultured in 96-well plates at a density of 5 × 10^5^ cells/well for 24 h. After treatment with **3** at the dose of 50 μmol/L, the total RNA was extracted using TRIzol^®^ reagent. Briefly, cells were collected, washed with 1 × PBS, lysed with TRIzol, and separated into phases by adding chloroform. The aqueous phase containing RNA was extracted, precipitated with isopropanol, washed with ethanol, and dissolved in RNase-free water. RNA concentration and purity were measured using a spectrophotometer. For cDNA synthesis, RNA samples were reverse-transcribed using a HiScript III RT SuperMix kit (Vazyme Biotech, Nanjing, China). RT-qPCR was performed with ChamQ Universal SYBR qPCR Master Mix on a Real-Time PCR Detection System. Primers for target genes (Bcl-2, Bax, caspase-3, Fas, FasL, and caspase-8) and the reference gene *β*-actin were designed based on GenBank sequences and synthesized. The primer sequences used in the RT-qPCR analysis are listed in Table S1. Gene expression levels were normalized to *β*-actin and calculated using the 2−ΔΔCt method.

## 4. Conclusions

The results of this study on *Serratia* sp. F2-2 demonstrated the potential of mongrove-derived bacteria as a source of novel bioactive compounds. Two new catechol siderophores, serratiochelins E (**1**) and F (**2**), were identified, expanding the known chemical diversity of serratiochelins. Most importantly, serratiochelin B (**3**) showed selective cytotoxicity against HepG2 liver cancer cells. Serratiochelin B also inhibited cell proliferation and induced apoptosis, as evidenced by morphological changes and increased chromatin condensation. The mechanism of action involved the modulation of apoptosis-related proteins. Specifically, it upregulated the Bax/Bcl-2 ratio and activated caspase-3, indicating mitochondrial pathway involvement. Additionally, it activated the Fas/FasL death receptor pathway, as shown by the increased FasL and caspase-8 expression. These findings suggest that serratiochelin B could serve as a lead compound for developing anticancer agents targeting liver cancer.

## Figures and Tables

**Table 1:** ^1^H (500 MHz) and ^13^C (125 MHz) NMR data for **1** and **2.**

No.	1 ^a^		2 ^b^	
	*δ*_C_, Type	*δ*_H_ (Mult., J in Hz)	*δ*_C_, Type	*δ*_H_ (Mult., J in Hz)
1	120.6 s		104.0 s	
2	148.2 s		151.7 s	
3	146.8 s		140.4 s	
4	121.8 d	7.05 (d, *J* = 7.5 Hz, 1H)	151.0 s	
5	123.6 d	6.97 (t, *J* = 7.5 Hz, 1H)	118.4 d	6.43 (d, overlapped, 1H)
6	119.6 d	7.38 (d, *J* = 7.5 Hz, 1H)	100.8 d	6.42 (d, *J* = 8.9 Hz, 1H)
7	165.3 s		169.8 s	
8				9.18 (d, *J* = 7.4 Hz, 1H)
9	62.2 d	5.08 (d, *J* = 5.0 Hz, 1H)	59.2 d	4.29 (dd, *J* = 7.4, 3.3 Hz, 1H)
10	171.7 s		169.5 s	
11				8.07 (t, *J* = 5.3 Hz, 1H)
12	37.7 t	3.31 (m, overlapped, 2H)	36.5 t	3.16 (m, 2H)
13	30.2 t	1.84 (m, 2H)	29.0 t	1.84 (m, 2H)
14	37.8 t	3.44 (t, *J* = 6.4 Hz, 2H)	36.6 t	3.30 (m, overlapped, 2H)
15				8.86 (br s, 1H)
16	171.7 s		169.8 s	
17	116.7 s		114.9 s	
18	150.3 s		149.9 s	
19	147.4 s		146.3 s	
20	119.7 d	6.98 (d, *J* = 7.8 Hz, 1H)	118.6 d	6.89 (d, *J* = 8.3 Hz, 1H)
21	119.6 d	6.71 (t, overlapped, 1H)	117.7 d	6.65 (t, *J* = 7.9 Hz, 1H)
22	118.6 d	7.22 (d, *J* = 7.9 Hz, 1H)	117.1 d	7.24 (d, *J* = 8.0 Hz, 1H)
23	67.8 d	4.44 (m, 1H)	66.3 d	4.13 (m, 1H)
24	20.7 q	1.27 (d, *J* = 6.1Hz, 3H)	20.4 q	1.10 (d, *J* = 6.3 Hz, 3H)
25	78.3 t	5.64 (d, *J* = 9.0 Hz, 1H) 5.55 (d, *J* = 9.0 Hz, 1H)	56.6 q	3.85 (s, 1H)

^a^ Data were taken in CD_3_OD; ^b^ Data were taken in DMSO-*d*_6_.

## Data Availability

The data presented in this research are available in this article and the Supplementary Materials.

## Supplementary Materials

The following supporting information can be downloaded at https://www.mdpi.com/article/10.3390/md23060241/s1: Figure S1: Strain information of *Serratia marcescens* F2-2; Figures S2–S7: The HR-ESI-MS, ^1^H NMR, ^13^C NMR and DEPT 135, ^1^H-^1^H COSY, HMQC, and HMBC spectra of **1**; Figure S8: NMR calculation results of **1**; Figure S9: The DP4+ probability analysis results of **1**; Figures S10–S15: The HR-ESI-MS, ^1^H NMR, ^13^C NMR, ^1^H-^1^H COSY, HMQC, and HMBC spectra of **2**; Figure S17: Purities of **1**–**4** using the HPLC analysis; Figure S18. Fluorescence microscopy images of HepG2 lines stained with AO and PI; Table S1: Primer sequences used in the RT-qPCR analysis.
