# Supplementary material for: Catechol Siderophores from a Mangrove-Derived Bacteria Serratia marcescens F2-2 and Their Cytotoxic Activity
Source: Mar Drugs. 2025 May 30;23(6):241. doi: 10.3390/md23060241 (PMC12193787; doi:10.3390/md23060241)
Supplement: Supplementary file 1 [file marinedrugs-23-00241-s001.zip › marinedrugs-3656156-supplementary.pdf]

SUPPLEMENTARY MATERIAL

Catechol siderphores from a mangrove-derived bacteria *Serratia marcescens* F2-2 and their cytotoxic activity

Gang Zhang [a,b,1], Xunming Wang [a,b,1], Xingwang Zhang [c], Lin Ye [a,b], Longyang Ke [a,b], Shimin Fan [a,b], Xuan Hong [a,b], Guoqiang Li [d], Bingye Yang [a,b,*] and Lianzhong Luo [a,b,*]

[a] Engineering Research Center of Marine Biopharmaceutical Resource of Fujian Province University, Xiamen Medical College, Xiamen 361023, China

[b] Xiamen Key Laboratory of Marine Medicinal Natural Product Resources, Xiamen Medical College, Xiamen 361023, China

[c] State Key Laboratory of Microbial Technology, Shandong University, Qingdao 266237, China

[d] Key Laboratory of Marine Drugs, Ministry of Education, School of Medicine and Pharmacy, Ocean University of China, Qingdao 266003, China

[*] Correspondence:yby@xmmc.edu.cn (B.Y.), lzluo@xmu.edu.cn (L.L.); Tel.: +86-592-6365150;

[1] These authors contributed equally to this work.

List of Supplementary Material

Figure S1. The 16S rRNA gene sequence, colony morphology and phylogenetic tree of *Serratia marcescens* F2-2

Figure S2. HR-ESI-MS of serratiochelin E (**1**)

Figure S3. ^{1}H NMR spectrum (500 MHz, CD_3OD) of serratiochelin E (**1**)

Figure S4. ^{13}C NMR and DEPT 135 spectra (125 MHz, CD_3OD) of serratiochelin E (**1**)

Figure S5. ^{1}H-^{1}H COSY spectrum (500 MHz, CD_3OD) of serratiochelin E (**1**)

Figure S6. HMQC spectrum (500 MHz, CD_3OD) of serratiochelin E (**1**)

Figure S7. HMBC spectrum (500 MHz, CD_3OD) of serratiochelin E (**1**)

Figure S8. The NMR calculation results for serratiochelin E (**1**)

Figure S9. The DP4+ probability analysis results for serratiochelin E (**1**)

Figure S10. HR-ESI-MS of serratiochelin F (**2**)

Figure S11. ^{1}H NMR spectrum (500 MHz, DMSO-d_6) of serratiochelin F (**2**)

Figure S12. ^{13}C NMR spectrum (125 MHz, DMSO-d_6) of serratiochelin F (**2**)

Figure S13. ^{1}H-^{1}H COSY spectrum (500 MHz, DMSO-d_6) of serratiochelin F (**2**)

Figure S14. HMQC spectrum (500 MHz, DMSO-d_6) of serratiochelin F (**2**)

Figure S15. HMBC spectrum (500 MHz, DMSO-d_6) of serratiochelin F (**2**)

Figure S16. Marfey's analysis results for serratiochelins F (**2**) and B (**3**)

Figure S17. Purities of compounds **1–4** using the HPLC analysis

Figure S18. Fluorescence microscopy images of HepG2 lines stained with AO and PI

Table S1. Primer sequences used in the RT-qPCR analysis

A:

TCGAGCGGTAGCACAGGGGAGCTTGCTCCCTGGGTGACGAGCGGCGGACGGGTGAGTAATGTCTGGGAAACTGCCTGAT
GGAGGGGGATAACTACTGGAAACGGTAGCTAATACCGCATAACGTCGCAAGACCAAAGAGGGGGACCTTCGGGCCTCTT
GCCATCAGATGTGCCCAGATGGGATTAGCTAGTAGGTGGGGTAATGGCTCACCTAGGCGACGATCCCTAGCTGGTCTGA
GAGGATGACCAGCCACACTGGAACTGAGACACGGTCCAGACTCCTACGGGAGGCAGCAGTGGGGAATATTGCACAATGG
GCGCAAGCCTGATGCAGCCATGCCGCGTGTGTGAAGAAGGCCTTCGGGTTGTAAAGCACTTTCAGCGAGGAGGAAGGTG
GTGAGCTTAATACGtTCATCAATTGACGTTACTCGCAGAAGAAGCACCGGCTAACTCCGTGCCAGCAGCCGCGGTAATA
CGGAGGGTGCAAGCGTTAATCGGAATTACTGGGCGTAAAGCGCACGCAGGCGGTTTGTTAAGTCAGATGTGAAATCCCC
GGGCTCAACCTGGGAACTGCATTTGAAACTGGCAAGCTAGAGTCTCGTAGAGGGGGGTAGAATTCCAGGTGTAGCGGTG
AAATGCGTAGAGATCTGGAGGAATACCGGTGGCGAAGGCGGCCCCCTGGACGAAGACTGACGCTCAGGTGCGAAAGCGT
GGGGAGCAAACAGGATTAGATACCCTGGTAGTCCACGCTGTAAACGATGTCGATTTGGAGGTTGTGCCCTTGAGGCGTG
GCTTCCGGAGCTAACGCGTTAAATCGACCGCCTGGGGAGTACGGCCGCAAGGTTAAAACTCAAATGAATTGACGGGGGC
CCGCACAAGCGGTGGAGCATGTGGTTTAATTCGATGCAACGCGAAGAACCTTACCTACTCTTGACATCCAGAGAACTTT
CCAGAGATGGATTGGTGCCTTCGGGAACTCTGAGACAGGTGCTGCATGGCTGTCGTCAGCTCGTGTTGTGAAATGTTGG
GTTAAGTCCCGCAACGAGCGCAACCCTTATCCTTTGTTGCCAGCGGTTCGGCCGGGAACTCAAAGGAGACTGCCAGTGA
TAAACTGGAGGAAGGTGGGGATGACGTCAAGTCATCATGGCCCTTACGAGTAGGGCTACACACGTGCTACAATGGCGTA
TACAAAGAGAAGCGACCTCGCGAGAGCAAGCGGACCTCATAAAGTACGTCGTAGTCCGGATTGGAGTCTGCAACTCGAC
TCCATGAAGTCGGAATCGCTAGTAATCGTAGATCAGAATGCTACGGTGAATACGTTCCCGGGCCTTGTACACACCGCCC
GTCACACCATGGGAGTGGGTTGCAAAAGAAGTAGGTAGCTTAACCTTCGGGAG

B:

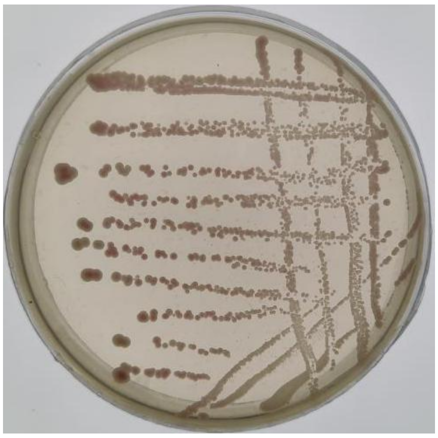

C:

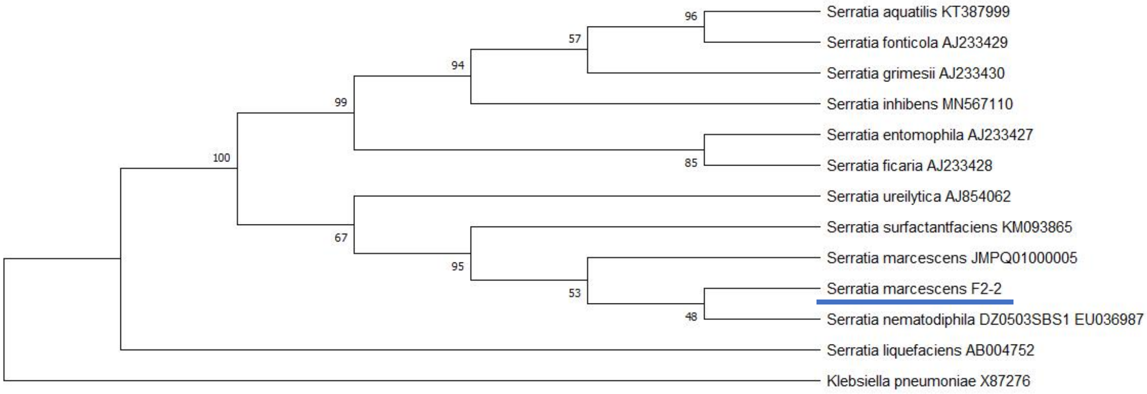

Figure S1. The 16S rRNA gene sequence, colony morphology and phylogenetic tree of *Serratia marcescens* F2-2

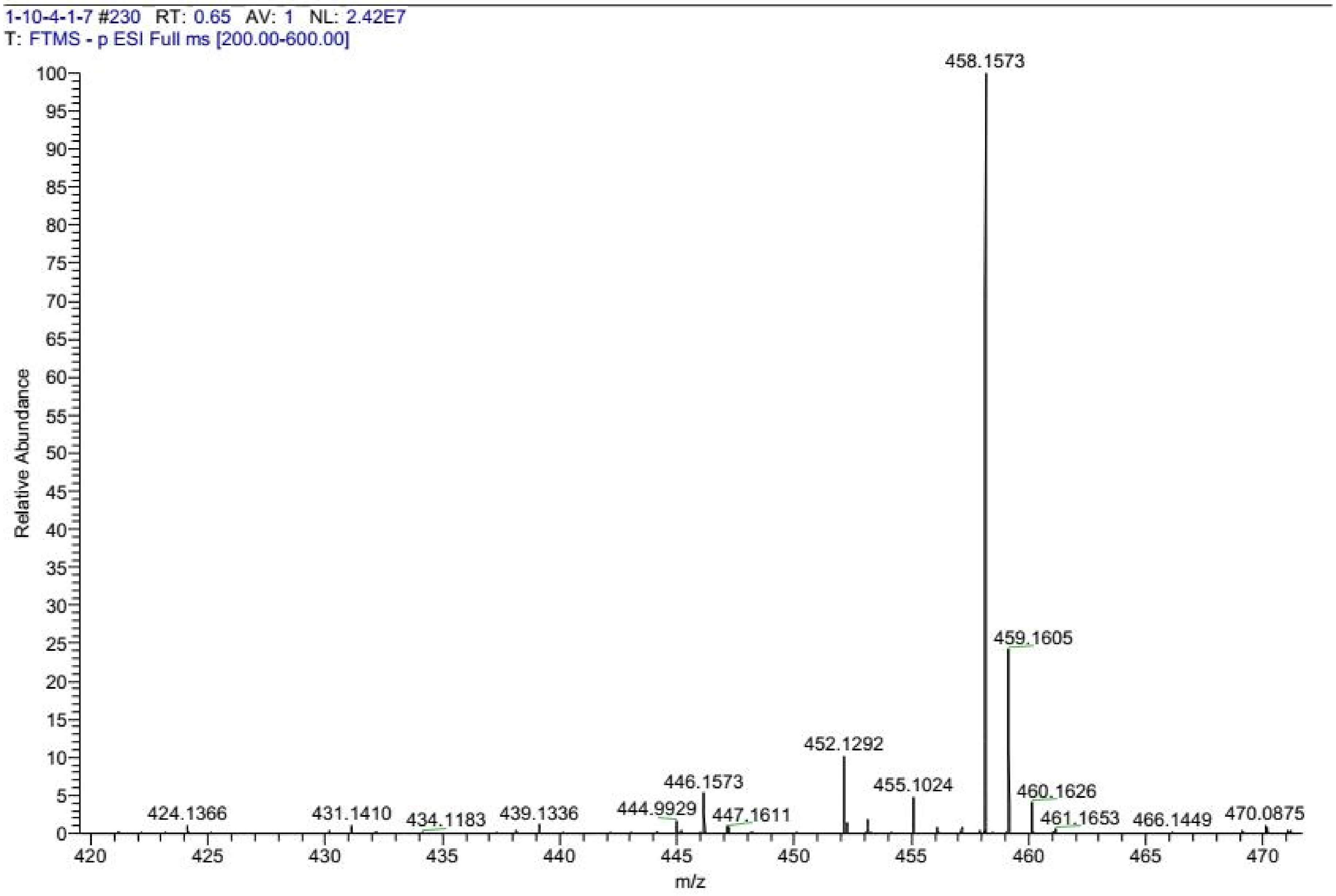

Figure S2. HR-ESI-MS of serratiochelin E (**1**).

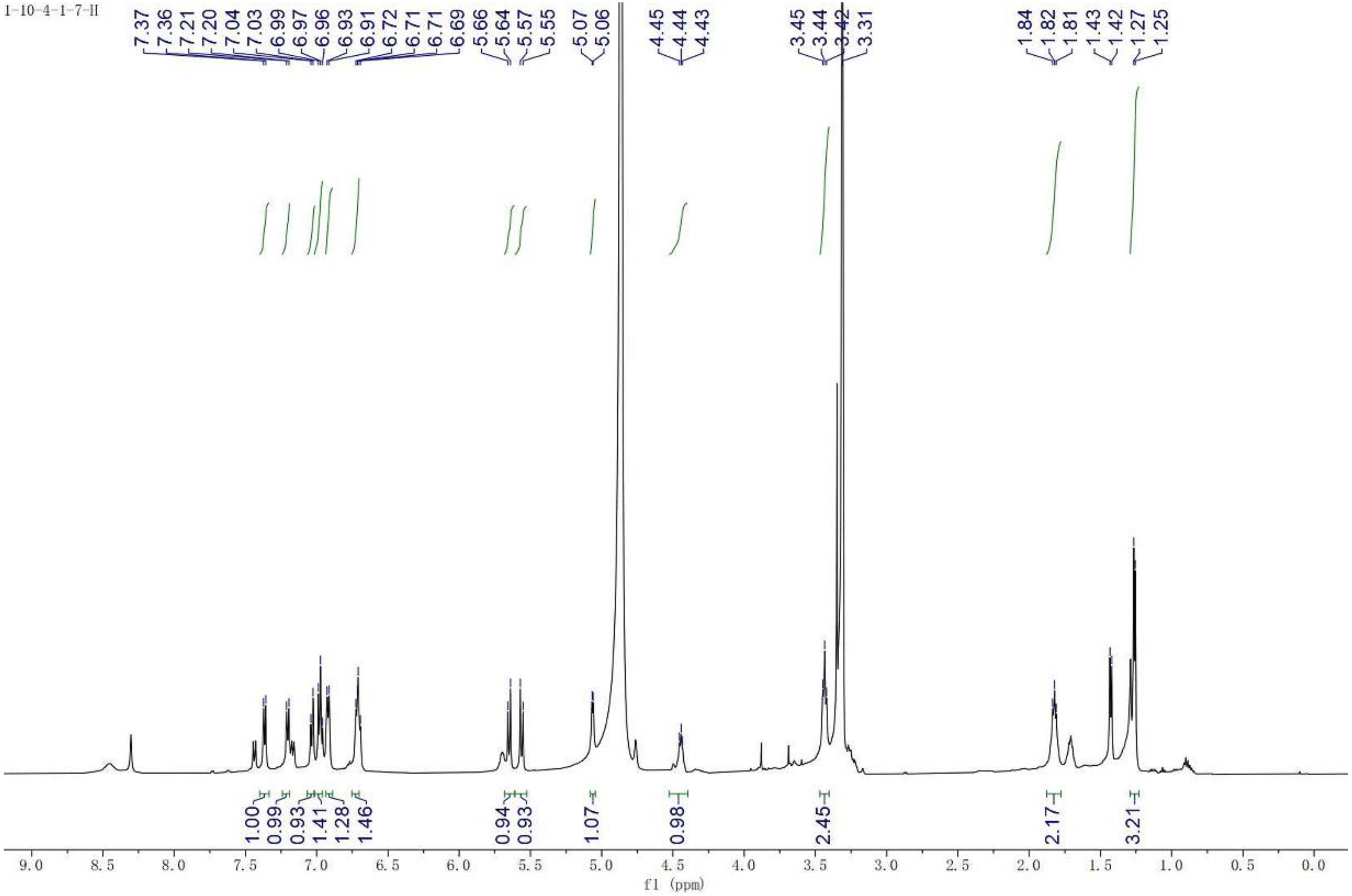

Figure S3. ^{1}H NMR spectrum (500 MHz, CD_3OD) of serratiochelin E (**1**)

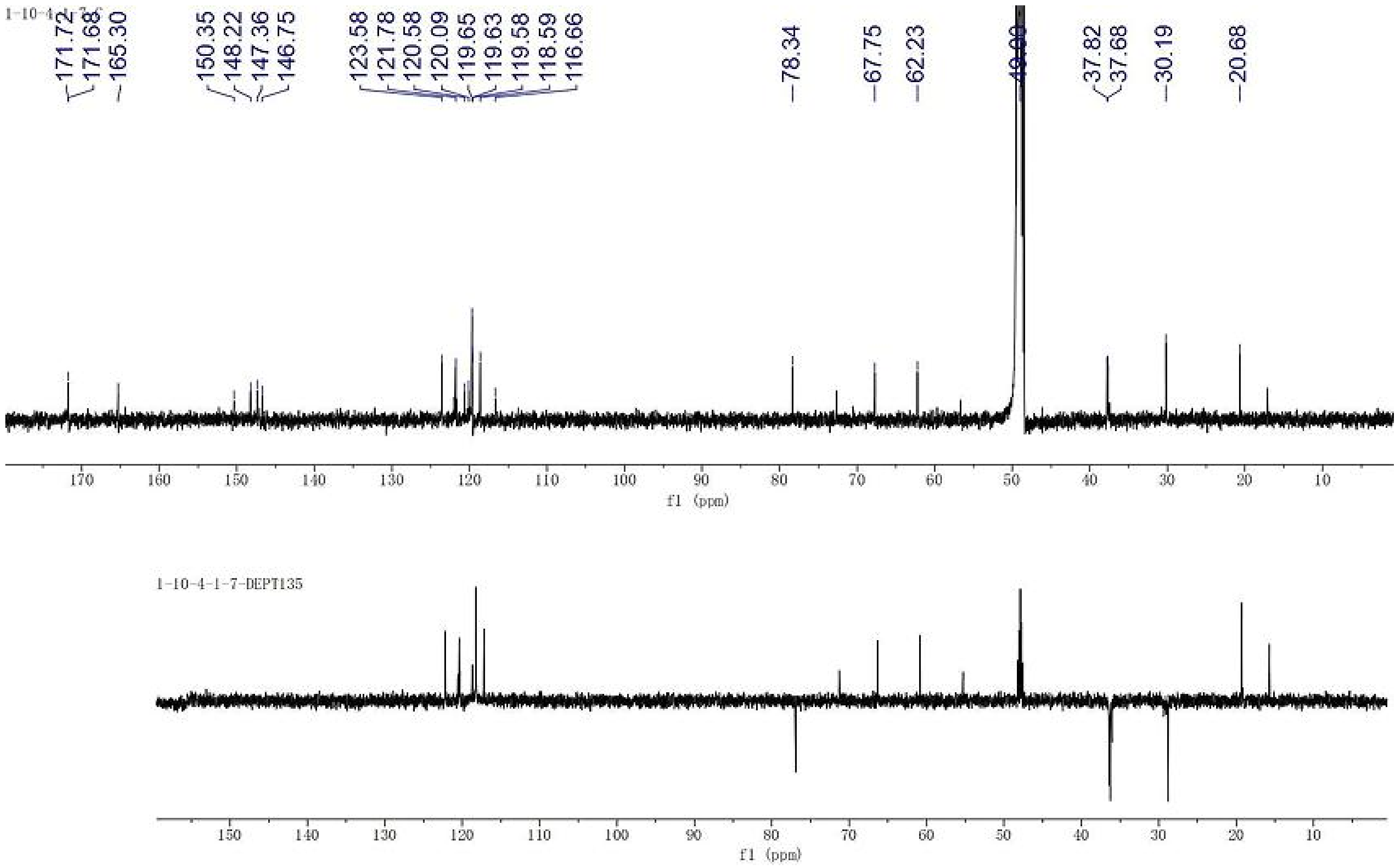

Figure S4. ^{13}C NMR and DEPT 135 spectra (125 MHz, CD_3OD) of serratiochelin E (**1**)

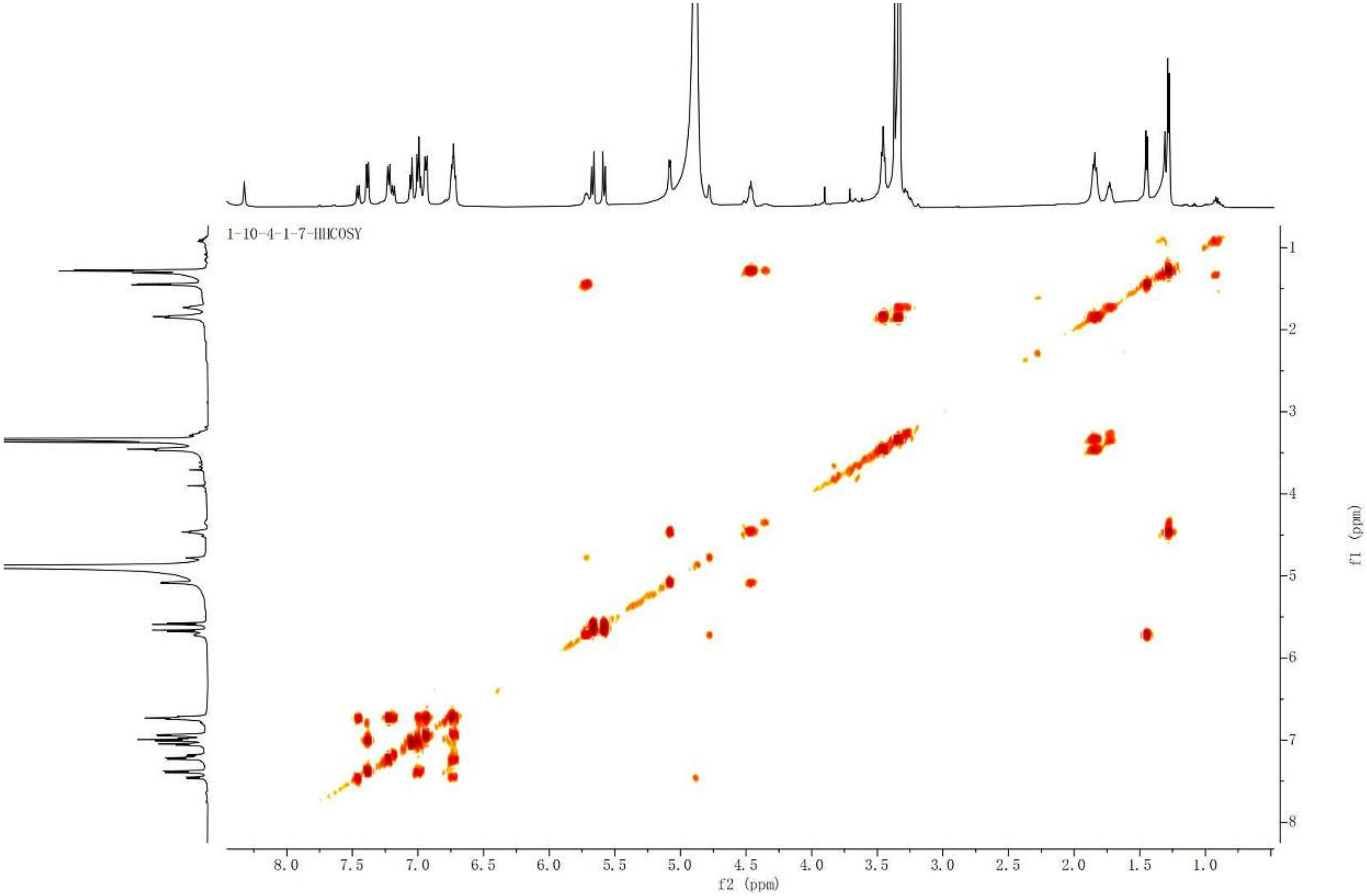

Figure S5. ^{1}H-^{1}H COSY spectrum (500 MHz, CD_3OD) of serratiochelin E (**1**)

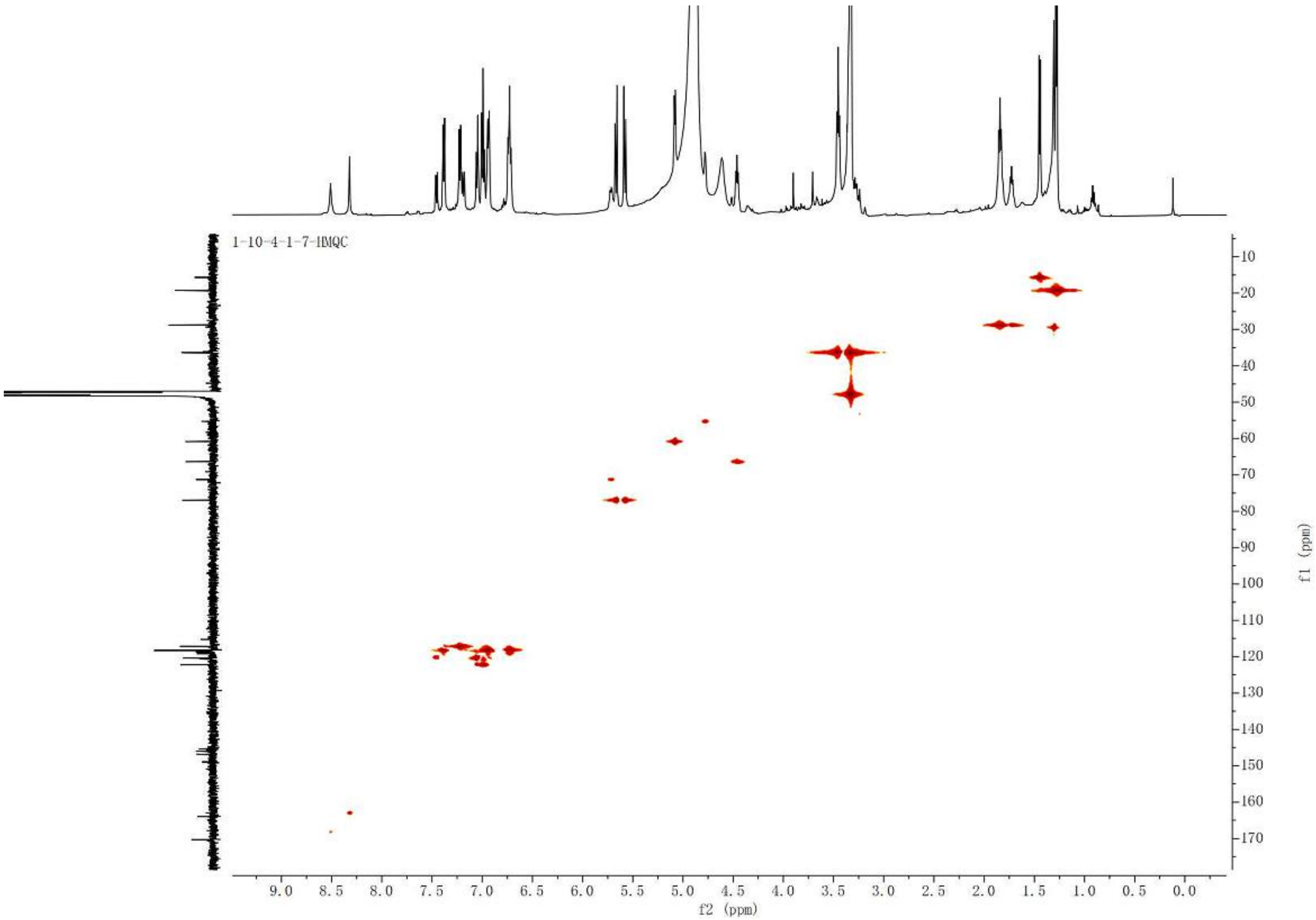

Figure S6. HMQC spectrum (500 MHz, CD_3OD) of serratiochelin E (**1**)

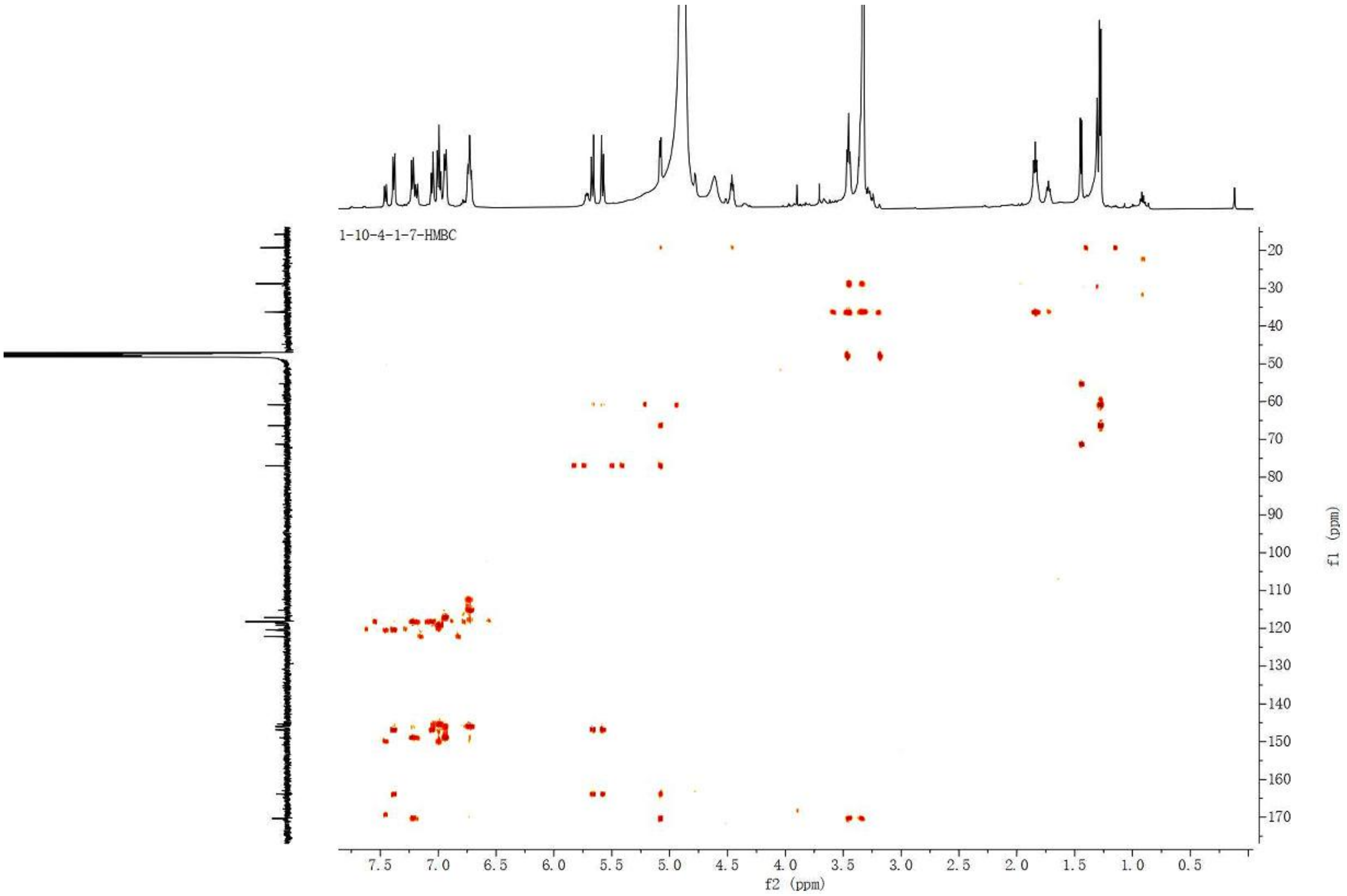

Figure S7. HMBC spectrum (500 MHz, CD_3OD) of serratiochelin E (**1**)

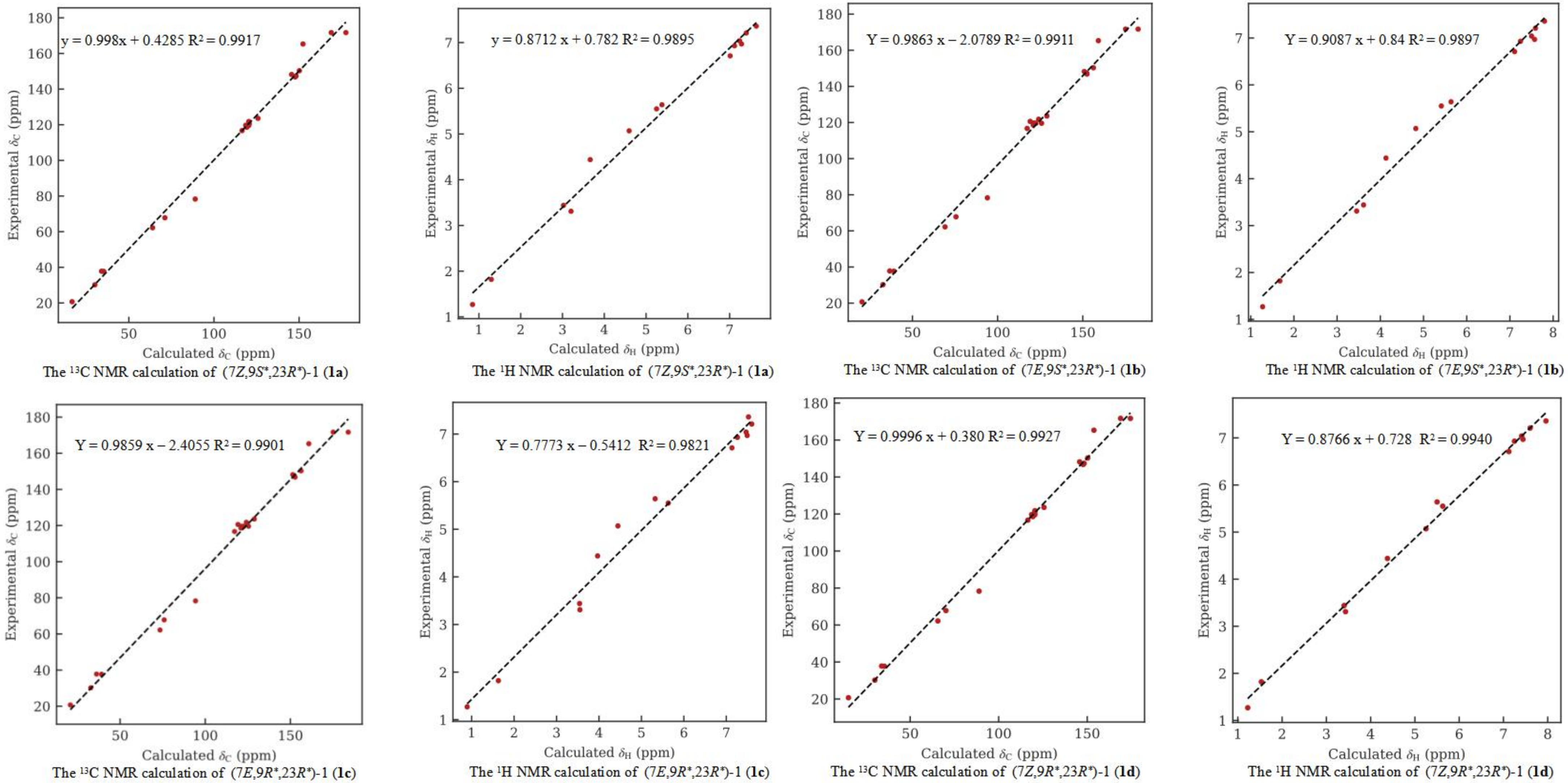

Figure S8. The NMR calculation results of serratiochelin E (**1**)

		DP4+	0.11%	0.00%	0.00%	99.89%
Nuclei	sp2?	xperimenta	Isomer 1	Isomer 2	Isomer 3	Isomer 4
C		120.6	120.8703408	115.3852076	115.109839	120.6635752
C		148.2	145.7376919	146.7379819	146.8438312	146.0450823
C		146.8	147.8316942	148.3157263	148.1794565	148.0311769
C		121.8	120.6533757	120.3209662	119.9325634	120.8154136
C		123.6	126.0335906	125.0839829	124.5713153	125.9207432
C		119.6	119.5891091	122.0156757	121.220766	119.773636
C		165.3	152.4125116	154.8982498	156.1333631	154.1085124
C		62.2	64.22359452	66.08753564	69.9682272	65.99584429
C		171.7	177.5738732	177.9089798	178.9505996	174.7128568
C		37.7	35.62242824	36.48731407	36.16814182	35.79459165
C		30.2	30.42175348	30.12586852	29.88635492	30.42326986
C		37.8	34.31614681	34.05586568	33.2329605	34.23592373
C		171.7	168.9903797	170.6564409	170.2206757	169.1352187
C		116.7	116.6990026	113.6387228	113.1474646	116.8064393
C		150.3	150.2992478	151.9847777	151.6377668	150.5958369
C		147.4	148.3705141	148.1324327	147.7632999	148.5741567
C		119.7	118.8385138	117.4221787	117.1192418	118.9311777
C		119.6	120.6364097	118.7040307	118.3580498	120.7510397
C		118.6	119.4557764	117.24329	116.8201138	119.64049
C		67.8	71.45659529	72.45133203	72.40965997	70.55699463
C		20.7	17.04097651	18.00310059	18.09041814	15.53050924
C		78.3	89.2264739	90.64033982	90.5358909	89.2575111
H		6.93	6.981920623	6.923009637	6.984646838	6.954850032
H		6.71	6.89114374	6.799390168	6.870091595	6.86876316
H		7.21	7.227506066	7.238303485	7.284655573	7.207587766
H		3.44	3.417185617	3.622165795	3.68490569	3.34801169
H		1.82	1.911831351	1.862757386	1.987074536	1.797439834
H		3.31	3.574041449	3.476565472	3.691782554	3.420685842
H		5.07	4.7846322	4.721001195	4.486171255	4.993261574
H		4.44	3.973912747	4.09302039	4.061225431	4.319381949
H		1.27	1.520381454	1.498646696	1.334911212	1.5356434
H		5.64	5.469379128	5.461971262	5.264188583	5.265022578
H		5.55	5.356735644	5.257473956	5.538908206	5.412124179
H		7.36	7.435979272	7.428547249	7.216330602	7.477420143
H		6.97	7.127581801	7.220081949	7.188734413	7.0987957
H		7.04	7.087768907	7.15706536	7.166373513	7.061012154

	A	B	C	D	E	F	G	H
1	Functional		Solvent?		Basis Set		Type of Data	
2	mPW1PW91		PCM		6-31+G(d,p)		Shielding Tensors	
3								
4			Isomer 1	Isomer 2	Isomer 3	Isomer 4	Isomer 5	Isomer 6
5	sDP4+ (H data)		0.40%	0.25%	0.00%	99.34%	-	-
6	sDP4+ (C data)		19.91%	0.00%	0.00%	80.09%	-	-
7	sDP4+ (all data)		0.10%	0.00%	0.00%	99.90%	-	-
8	uDP4+ (H data)		25.03%	25.03%	24.90%	25.04%	-	-
9	uDP4+ (C data)		3.84%	30.63%	62.04%	3.50%	-	-
10	uDP4+ (all data)		3.85%	30.72%	61.92%	3.51%	-	-
11	DP4+ (H data)		0.40%	0.25%	0.00%	99.34%	-	-
12	DP4+ (C data)		21.43%	0.02%	0.00%	78.55%	-	-
13	DP4+ (all data)		0.11%	0.00%	0.00%	99.89%	-	-

Figure S9. The DP4+ probability analysis results of serratiochelin E (**1**)

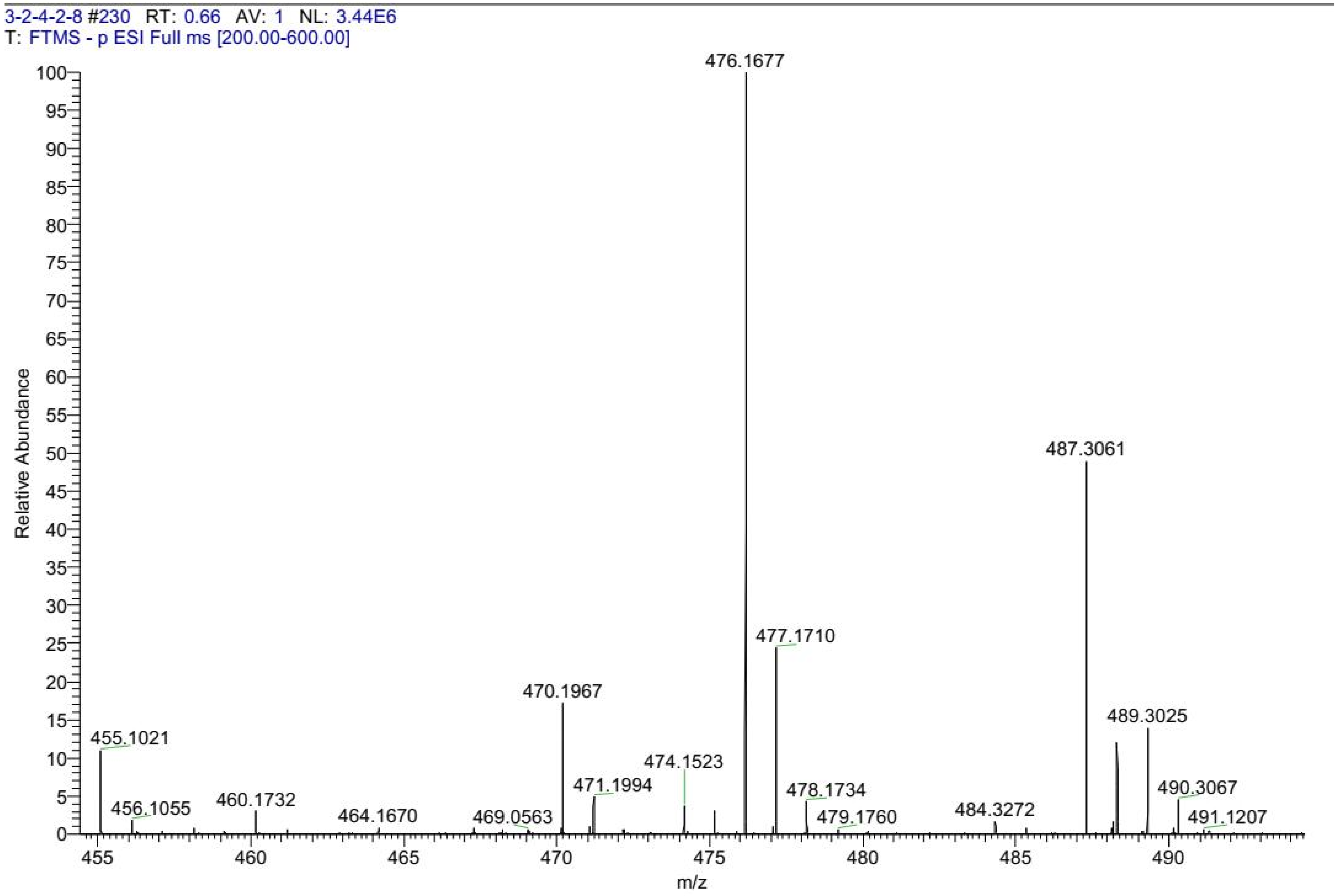

Figure S10. HR-ESI-MS of serratiochelin F (**2**)

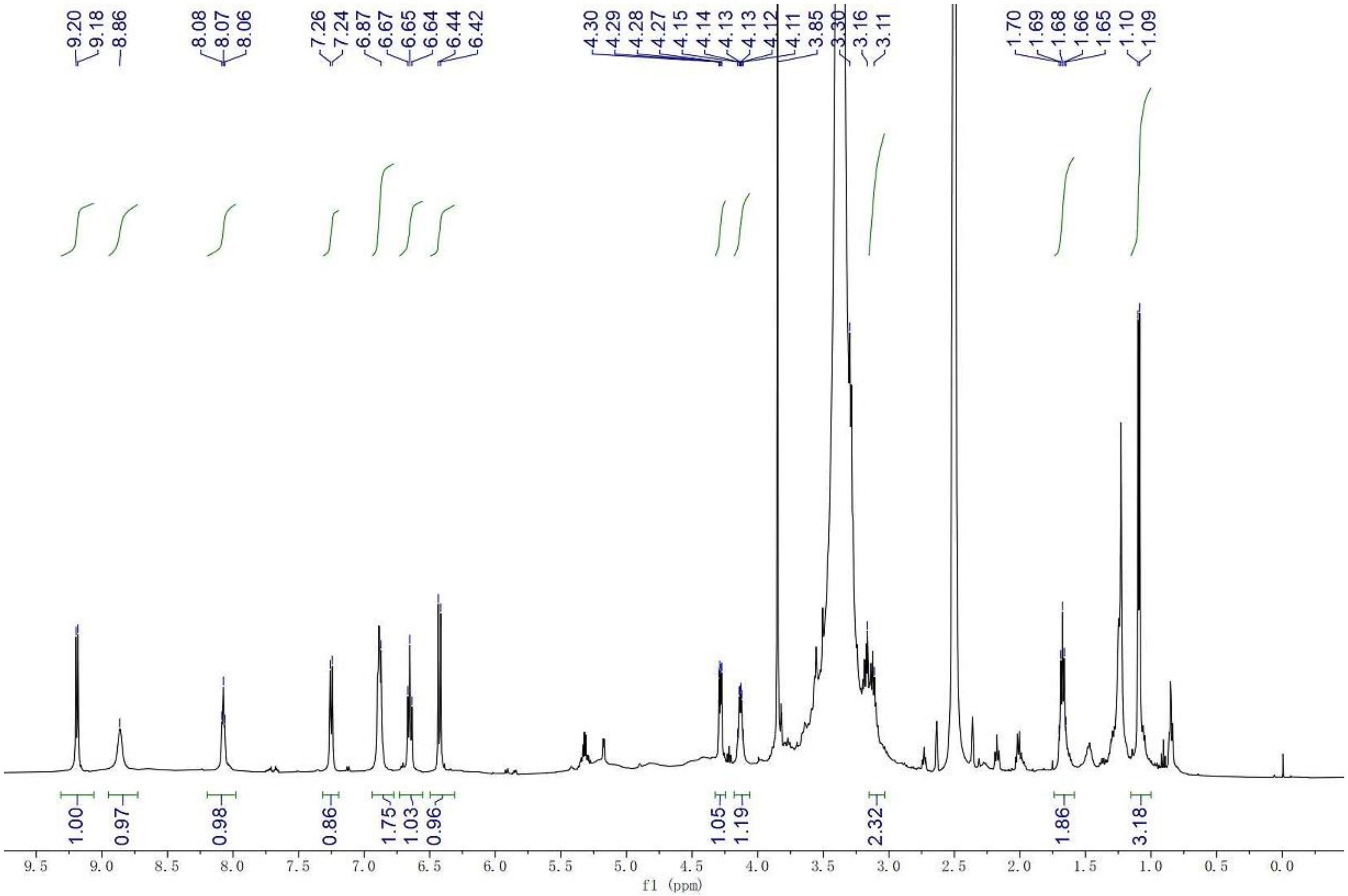

Figure S11. ^{1}H NMR spectrum (500 MHz, DMSO-d_6) of serratiochelin F (**2**)

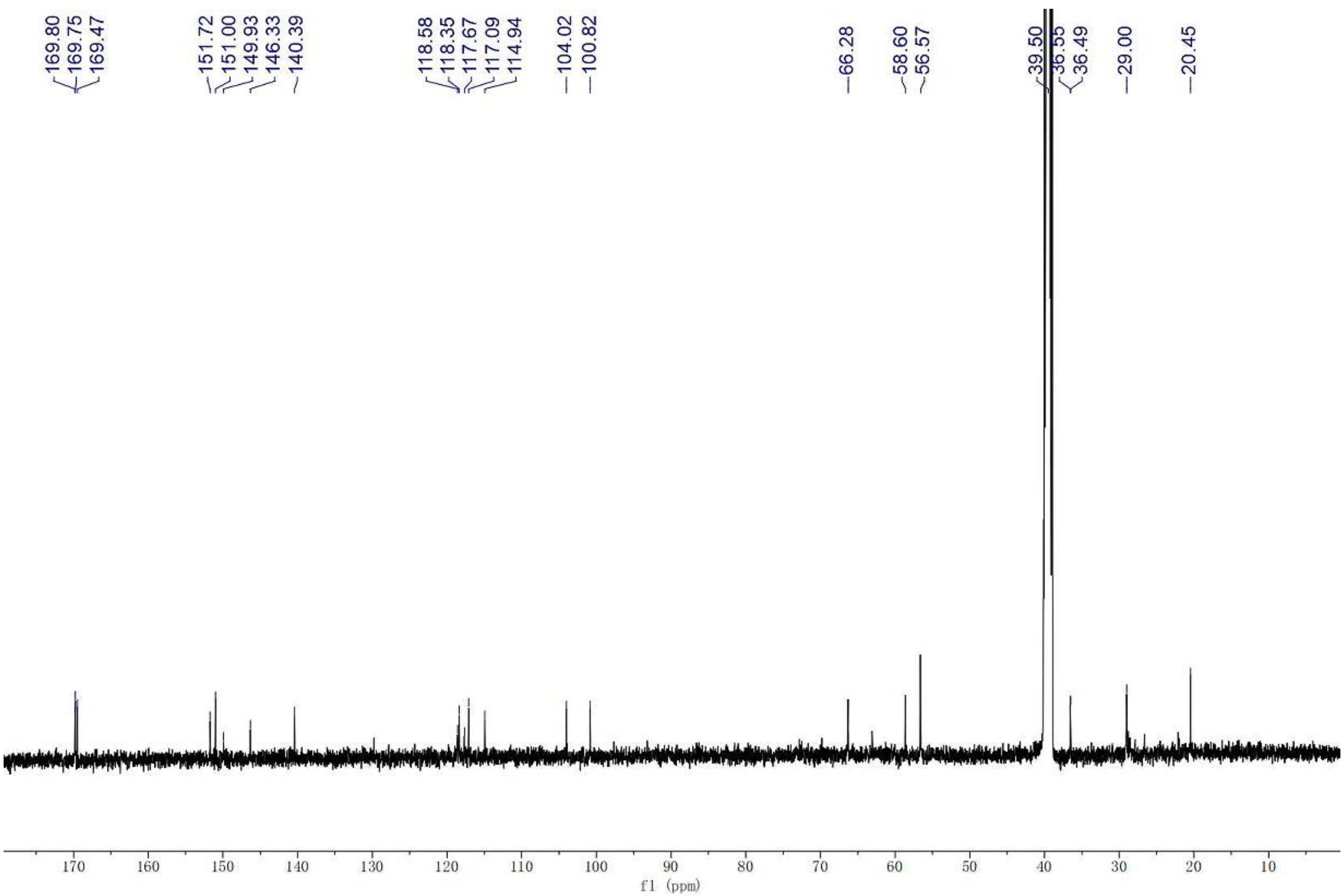

Figure S12. ^{13}C NMR spectrum (125 MHz, DMSO-d_6) of serratiochelin F (**2**)

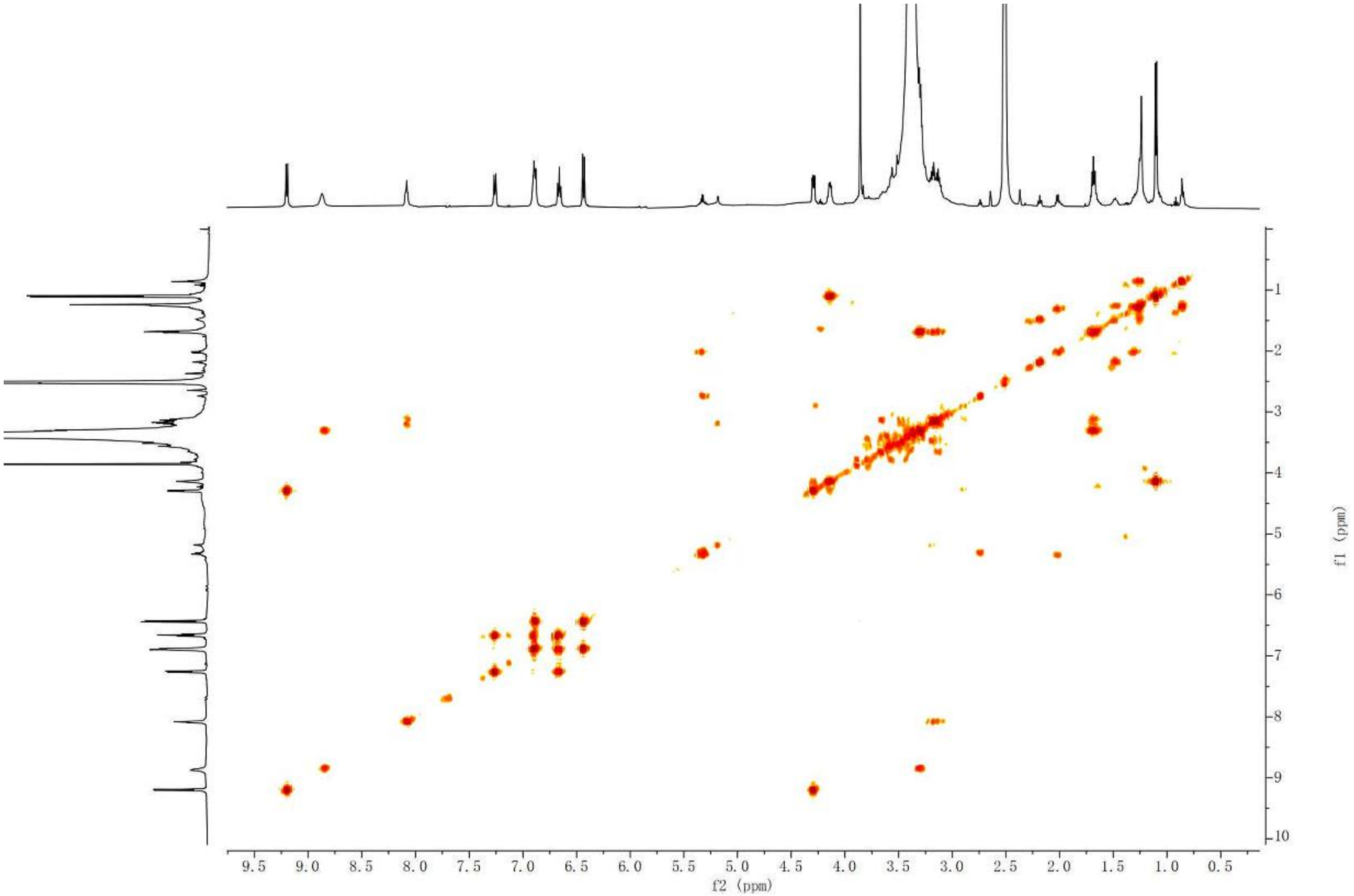

Figure S13. ^{1}H-^{1}H COSY spectrum (500 MHz, DMSO-d_6) of serratiochelin F (**2**)

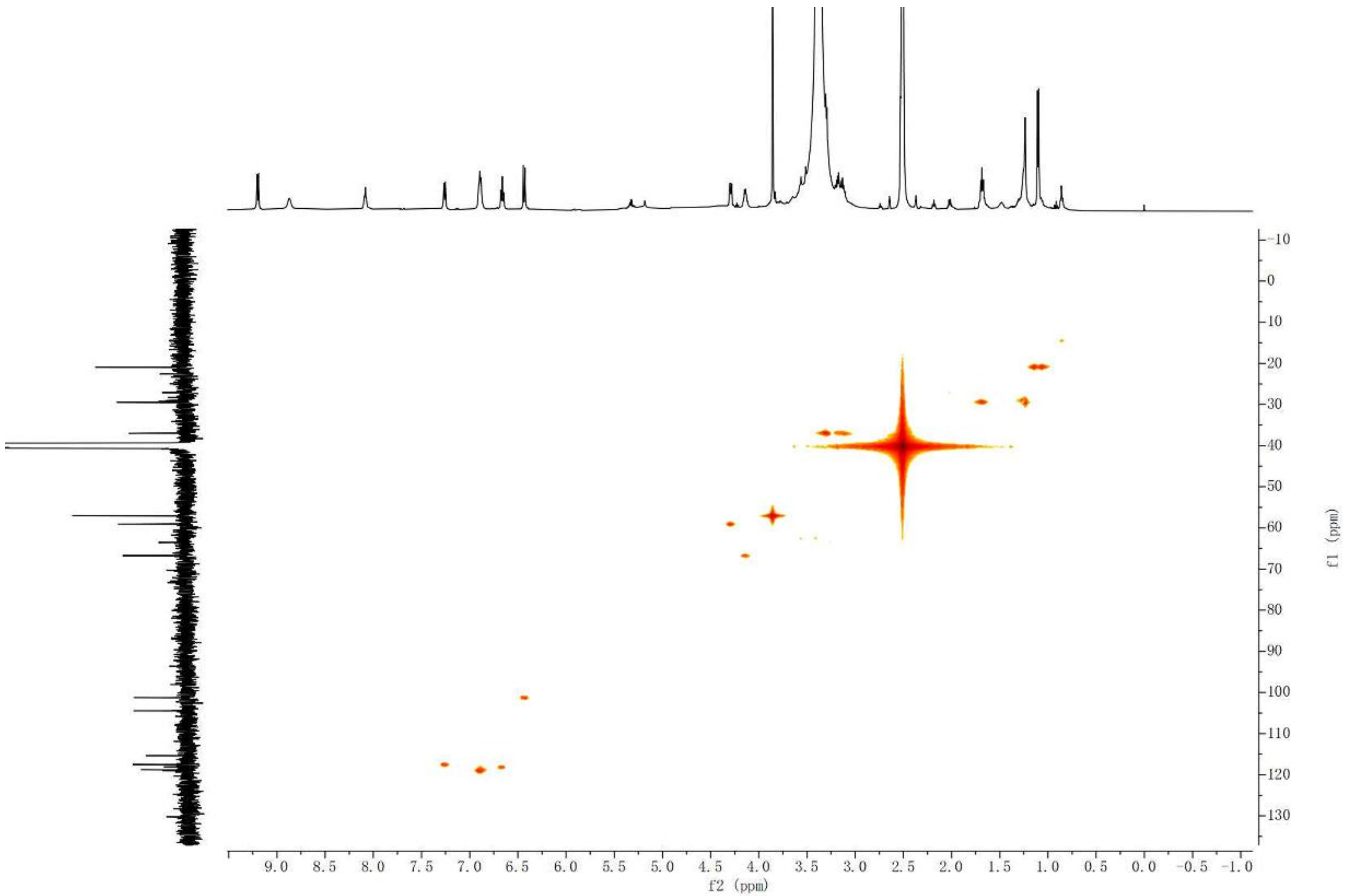

Figure S14. HMQC spectrum (500 MHz, DMSO-d_6) of serratiochelin F (**2**)

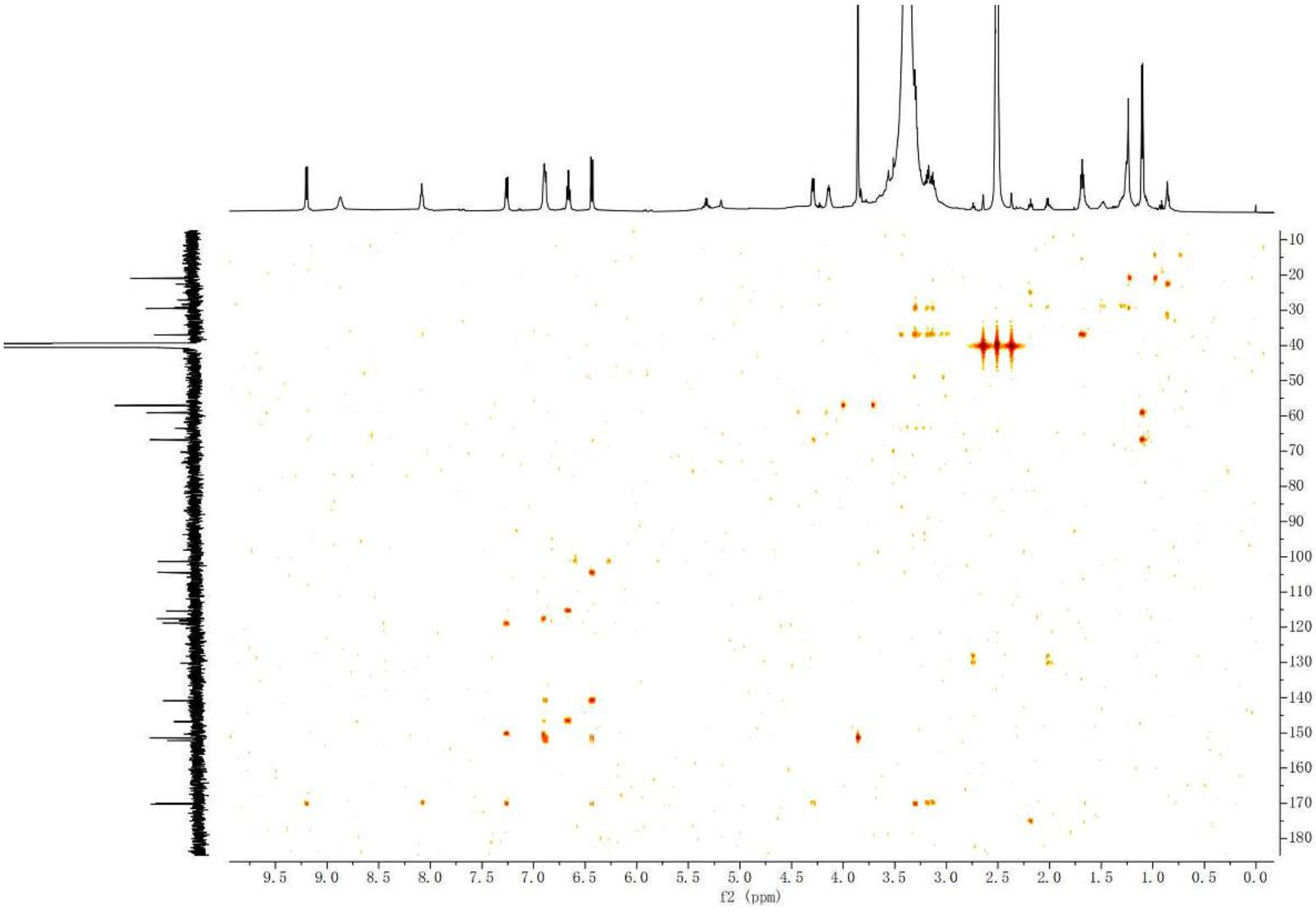

Figure S15. HMBC spectrum (500 MHz, DMSO-d_6) of serratiochelin F (**2**)

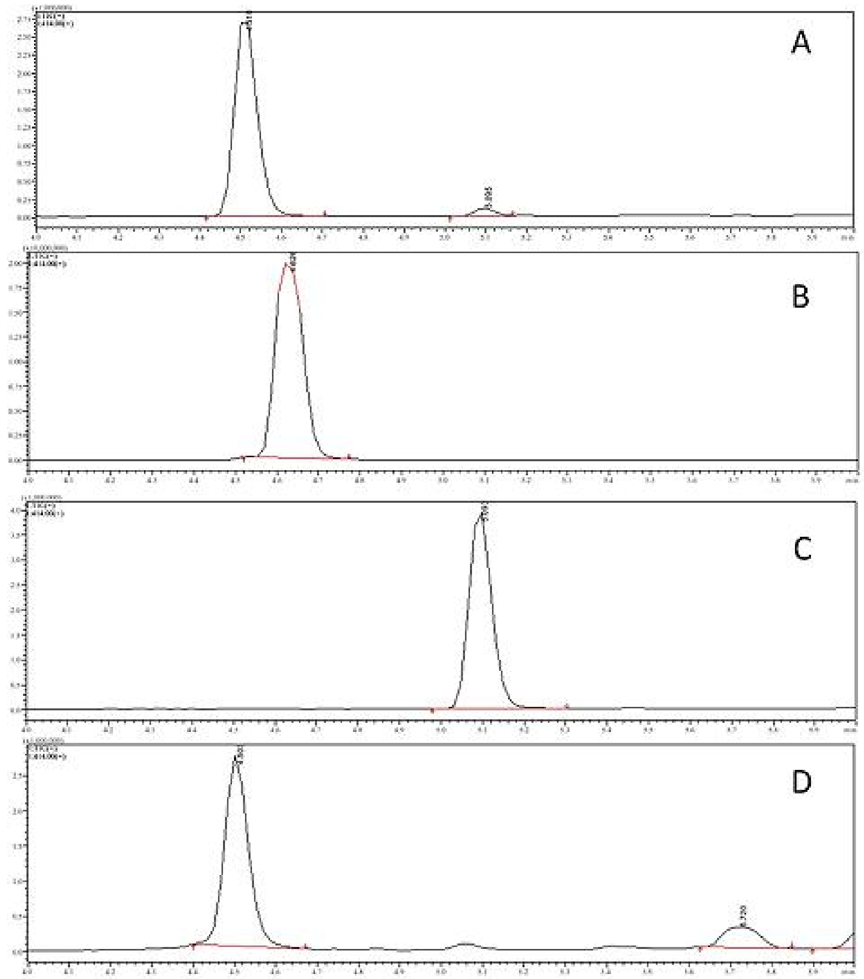

Figure S16. Marfey's analysis results for serratiochelins F (**2**)

A-D: FDLA derivatives of L-threonine, L-*allo*-threonine, D-threonine and the hydrolysates of **2**, respectively.

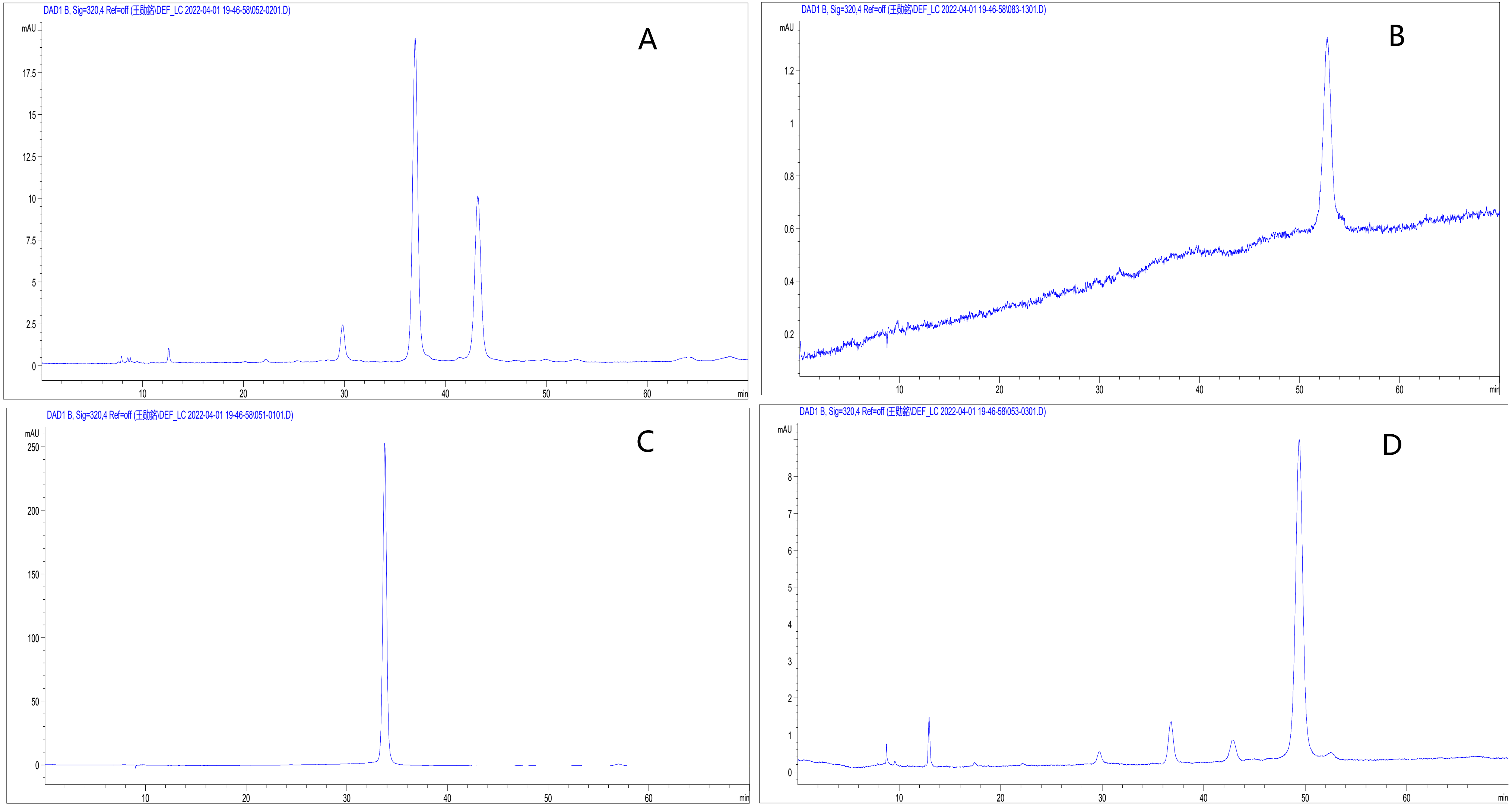

Figure S17. Purities of compounds **1**–**4** using the HPLC analysis A-D: serratiochelins E (**1**), F (**2**), B (**3**) and A (**4**), respectively. The separation condition was as follows: An agilent 1260 HPLC equipped with an C_{18} column (5 µm, 10 × 250 mm, Reprosil Gold 120, Germany) isocratic elution with acetonitrile-water (25% acetonitrile containing 0.1% formic acid) at the flow rate of 1.5 mL/min for 70 min.

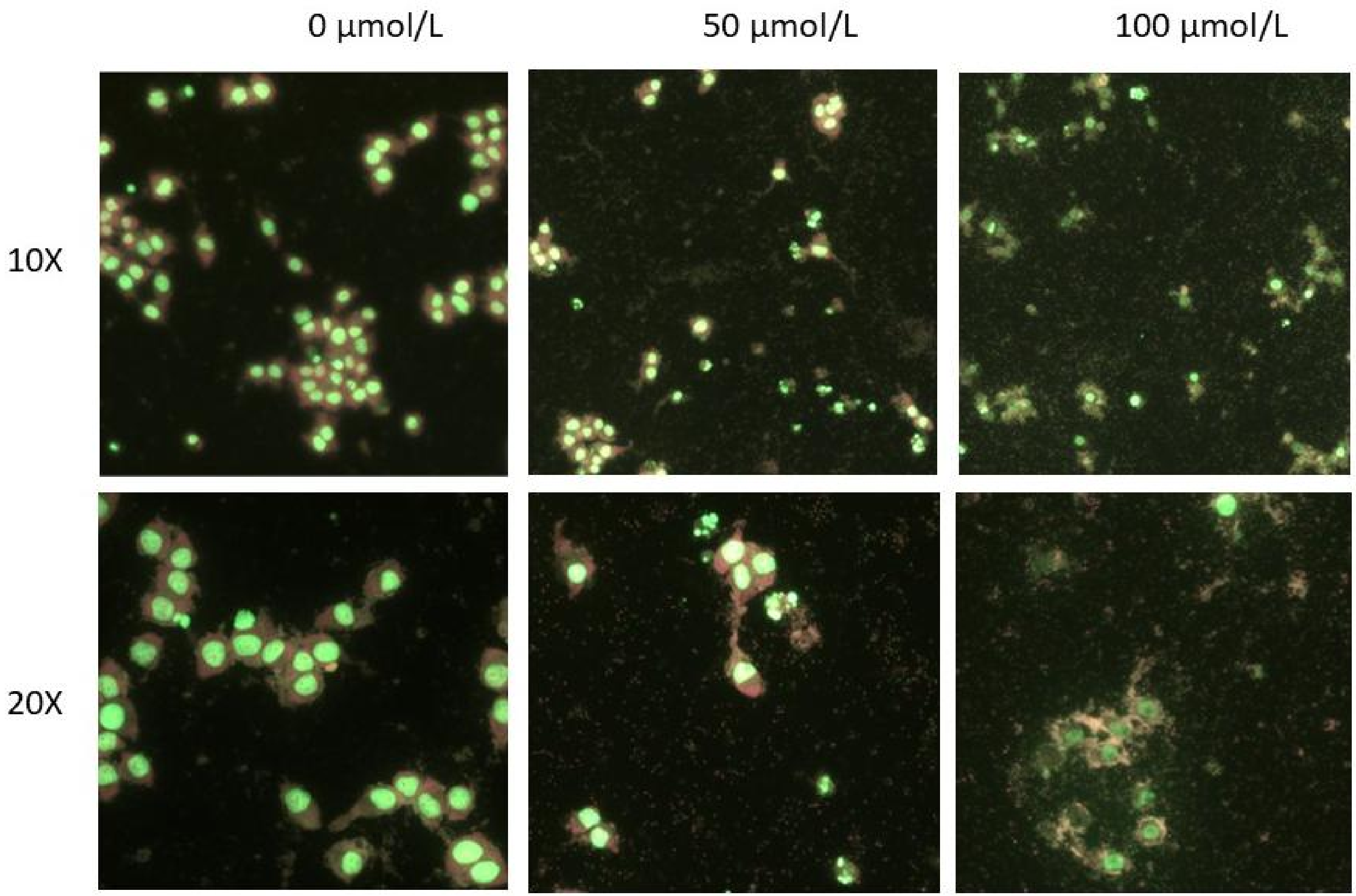

Figure S18. Fluorescence microscopy images of HepG-2 lines stained with AO and PI

Table S1. Primer sequences used in the RT-qPCR analysis

Primer Name	sequences
Fas	Forward：CAAGGGATTGGAATTGAGGA
	Reverse：TGGAAGAAAAATGGGCTTT
FasL	Forward：TGGCCTTGTGATCAATGAAA
	Reverse：TCATCATCTTCCCCTCCATC
caspase-8	Forward：AAGCAAACCTCGGGGATACT
	Reverse：GGGGCTTGATCTCAAAATGA
caspase-3	Forward：AAATACCAGTGGAGGCCGAC
	Reverse：ATGGCACAAAGCGACTGGAT
Bcl-2	Forward：ATCTCCCTGTTGACGCTCT
	Reverse：CATCTTCTCCTTCCAGCCT
Bax	Forward：TGCAGAGGATGATTGCTGAC
	Reverse：GAGGAVTCCAGCCACAAAGA
β-Actin	Forward：TCATCACTATTGGCAACGAGC
	Reverse：AACAGTCCGCCTAGAAGCAC
